# Anticancer properties of dried-pericarp water extracts of *Camellia japonica* L. fermented with *Aspergillus oryzae* through regulation of IGFBP-2/mTOR pathway

**DOI:** 10.1038/s41598-021-01127-3

**Published:** 2021-11-02

**Authors:** Eugene Cho, Jin Kim, Da Hye Jeong, Hyoun Woo Kim

**Affiliations:** 1 Jeollanam-Do Forest Resource Research Institute, Naju, Jeonnam 58213 Republic of Korea; 2 Gwangju Health University, Gwangsan-gu, Gwangju, 62287 Republic of Korea

**Keywords:** Cancer, Cell biology, Oncology

## Abstract

This study aimed to investigate the anticancer activity of dried-pericarp water extract of *fermented C. japonicus* (CJ). The dried-pericarp water extracts of CJ were fermented using *Aspergillus oryzae* and *Saccharomyces cerevisiae* at 30 °C and 35 °C. The anticancer activities of both water extracts fermented at 30 °C and 35 °C using *A. oryzae* against FaDu cells were remarkably changed compared with unfermented dried-pericarp water extract of CJ, which has no anticancer activity. Cleaved-PARP, caspase 3, and apoptotic cells stained with annexin V/PI were significantly increased by treatment with *A. oryzae* extracts fermented at 30 °C. The insulin-like growth factor-binding protein 2 (IGFBP-2) protein level and mTOR phosphorylation by *A. oryzae* fermented extracts (AOFE) were dramatically reduced, and the expression levels of IGFBP-2 and phosphorylated mTOR were significantly increased depending on the glucose concentrations in FaDu cells. These results suggested that the cell viabilities in AOFE were restored as the glucose concentrations increased. Furthermore, it was confirmed LC/MS/MS that the content of gallic acid was increased by fermentation of *Aspergillus oryzae* (5.596 ± 0.1746 μg/mg) compared to the unfermented extract (1.620 ± 0.0432 μg/mg). Based on these results, the anticancer effect of AOFE was achieved through inhibition of the IGFBP-2/mTOR signaling pathway. These results suggest that AOFE may be a potential treatment for head and neck cancer.

## Introduction

Cancer is a disease characterized by uncontrolled cell proliferation and is the leading cause of death worldwide^1,2^. Chemotherapy is one of the most common treatments for cancer; however, cancer cells may be inherently resistant to chemotherapy or develop resistance to drugs during treatment. Acquired or inherent resistance is a major cause of cancer-related deaths due to treatment failure in cancer patients^3,4^. Moreover, chemotherapeutic agents have cytotoxicity affects against normal cells^5,6^. Most chemotherapeutic and molecularly targeted agents induce apoptosis, but many types of cancers eventually become resistant to anticancer agents through evasion of apoptosis. Drug resistance caused by evasion of apoptosis is a major factor in the cancer cell survival, cancer recurrence, and limited effectiveness of chemotherapy^3,7,8^. Natural products are abundant sources of substances that can be used in formulating drugs. Approximately 25% of clinically used drugs are produced worldwide, and more than 60% of anticancer drugs are derived from plants^9,10^. Despite the numerous anticancer agents developed from plants, plant-derived metabolites remain a good source of developing new drugs with low cytotoxicity and increased activity^11,12^. Therefore, there is a need to discover new sources of more effective drugs and to reduce the side effects of chemotherapy in order to protect the normal cells.

*Camellia japonica* L. (named “dongbaek” in Korea) is a species of the genus *Camellia* and is widely distributed in Korea, China, and Japan. It has the following biological activities: antioxidant activity^13^, anti-inflammatory^14^, antiallergic responses^15^, antibacterial activity^16^, antimetastatic activity^17^, antiphotoaging^18^, antiatherogenic activities^19^, inhibition of human immunodeficiency virus type 1 protease^20^, and Epstein-Barr virus inhibition^21^. Previous studies investigating the biological activities of *C. japonica* L. were usually conducted using the leaf, flower, or oil of this plant. The fruit extracts of *C. japonica* (CJ) have been reported to have anticancer activity^22^, cardiovascular protection effect^23^, and gastroprotective effect via suppression of MAPK/NF-kB signaling pathways^24^, and to promote wound healing in a mouse wound model and enhance the generation of mouse and human iPSCs^25^. However, the number of studies assessing the physiological activity of CJ fruit remains insufficient.

IGFBP-2 is a member of the IGFBP family and exhibits various biological functions through IGF-dependent or IGF-independent mechanisms. IGFBP-2 binds to IGFs and therefore inhibits IGF signaling^26–28^. The overexpression of IGFBP-2 in human embryonic kidney fibroblasts inhibits cell proliferation, which is compensated by the addition of exogenous IGFs^26^. Secreted and membrane-associated IGFBP-2 compete with the IGF receptors for ligand binding and consequently modulate IGF responsiveness in patients with lung cancer^27^. Membrane-associated IGFBP-2 promotes cell proliferation, migration, invasion, and apoptosis in various types of cancers^28–30^. Interestingly, the PI3K/AKT/mTOR signaling pathway is regulated by IGFBP-2, which is associated with increased expression of IGFBP-2 and loss of PTEN^31,32^.

IGFBP-2 serum levels were significantly higher in head and neck squamous cell carcinoma (HNSCC) patients than in healthy controls^33^. However, the role of IGFBP-2 in head and neck cancer remains unclear. This study aimed to investigate the potential of fermented CJ dried fruit extracts as a new source of cancer treatment, the role of IGFBP-2 overexpression in the development of head and neck cancer, and their mechanisms.

## Results

### Fermentation improving the anticancer effect of dried *C. japonica* pericarp

**Figure 1:**
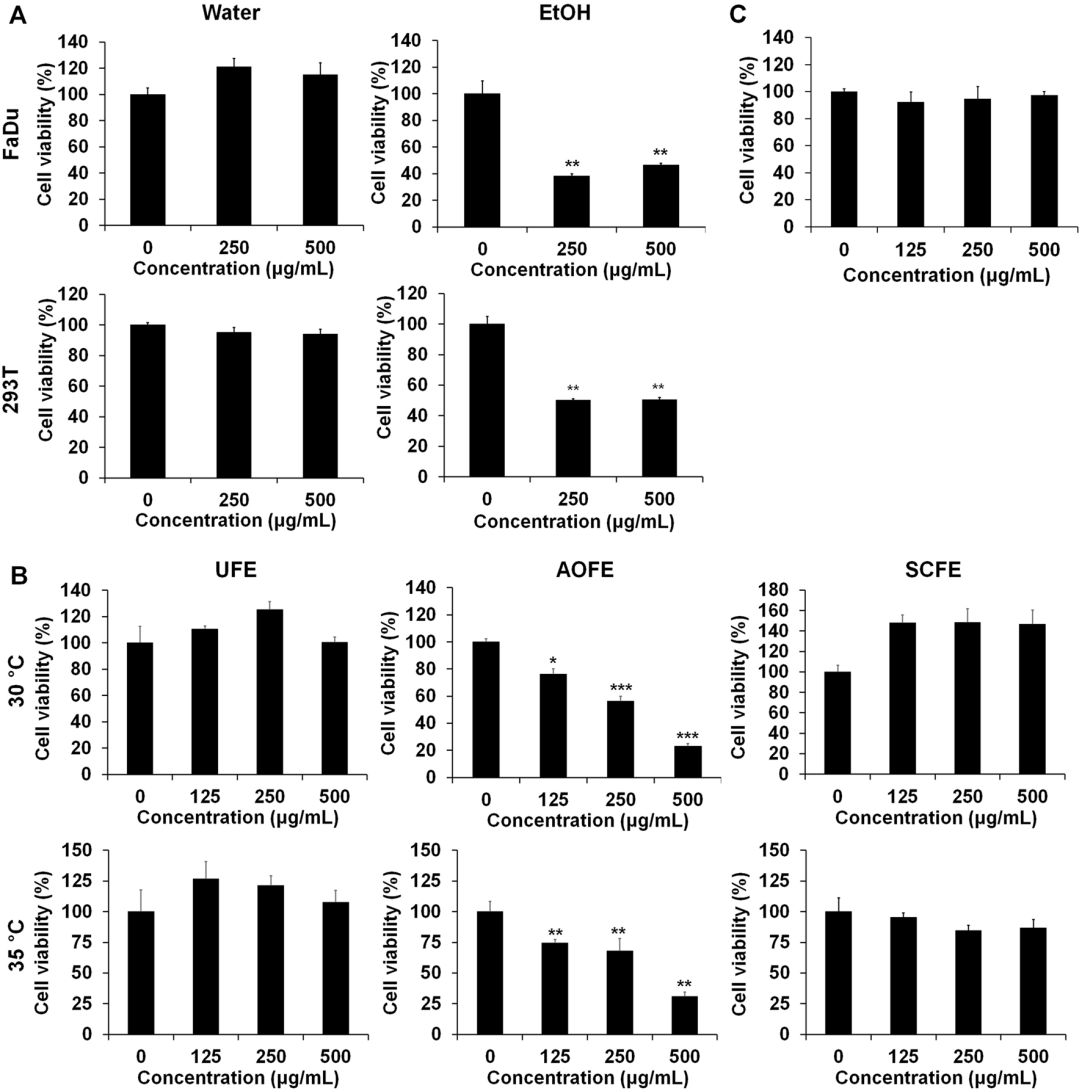
The effects of AOFE on the cell viability of head and neck cancer cells. (**A**) Head and neck cancer cells (FaDu) and normal cells (293 T) were plated and treated with water and EtOH extracts. (**B**) Powder of dried CJ pericarp was fermented at 30 °C or 35 °C for 3 days with *A. oryzae* (AOFE) and *S. cerevisiae* (SCFE). Fermented products were filtered and lyophilized and then treated to the cells. (**C**) The extract fermented at 30 °C with *A. oryzae* was treated with 293 T normal cells. Cell viability was measured by MTT assay. All data are presented as the mean ± SD. Statistical analyses were performed using paired t-test. *p < 0.05, **p < 0.01, ***p < 0.001.

We performed an MTT assay to evaluate the effect of water and ethanol extracts of dried pericarps on cell viability in the HNSCC cell line FaDu. Results showed that water extracts had no effect, but ethanol extracts significantly decreased the viability of FaDu cells. However, ethanol extracts also showed strong cytotoxicity in normal human embryonic kidney 293 T cells (Fig. 1A). Subsequently, in order to assess whether the fermented extract after extracting the dried pericarp with water can improve the anticancer effect, cell viability was analyzed by conducting an MTT assay after fermentation with *Aspergillus oryzae* (*A. oryzae*) and *Saccharomyces cerevisiae* (*S. cerevisiae*). Results showed that the cell viability was significantly decreased in *A. oryzae* extracts (AOFE) fermented at 30 °C and 35 °C compared with unfermented extracts (UFE). On the contrary, the fermentation of *S. cerevisiae* extracts (SCFE) did not improve its anticancer effect, but rather increased the cell viability at a fermentation temperature of 30 °C (Fig. 1B). *A. oryzae* extracts fermented at 30 °C had no effect on normal human embryonic kidney 293 T cells (Fig. 1C).

### The inhibitory effect of AOFE on cell viability is due to apoptosis

**Figure 2:**
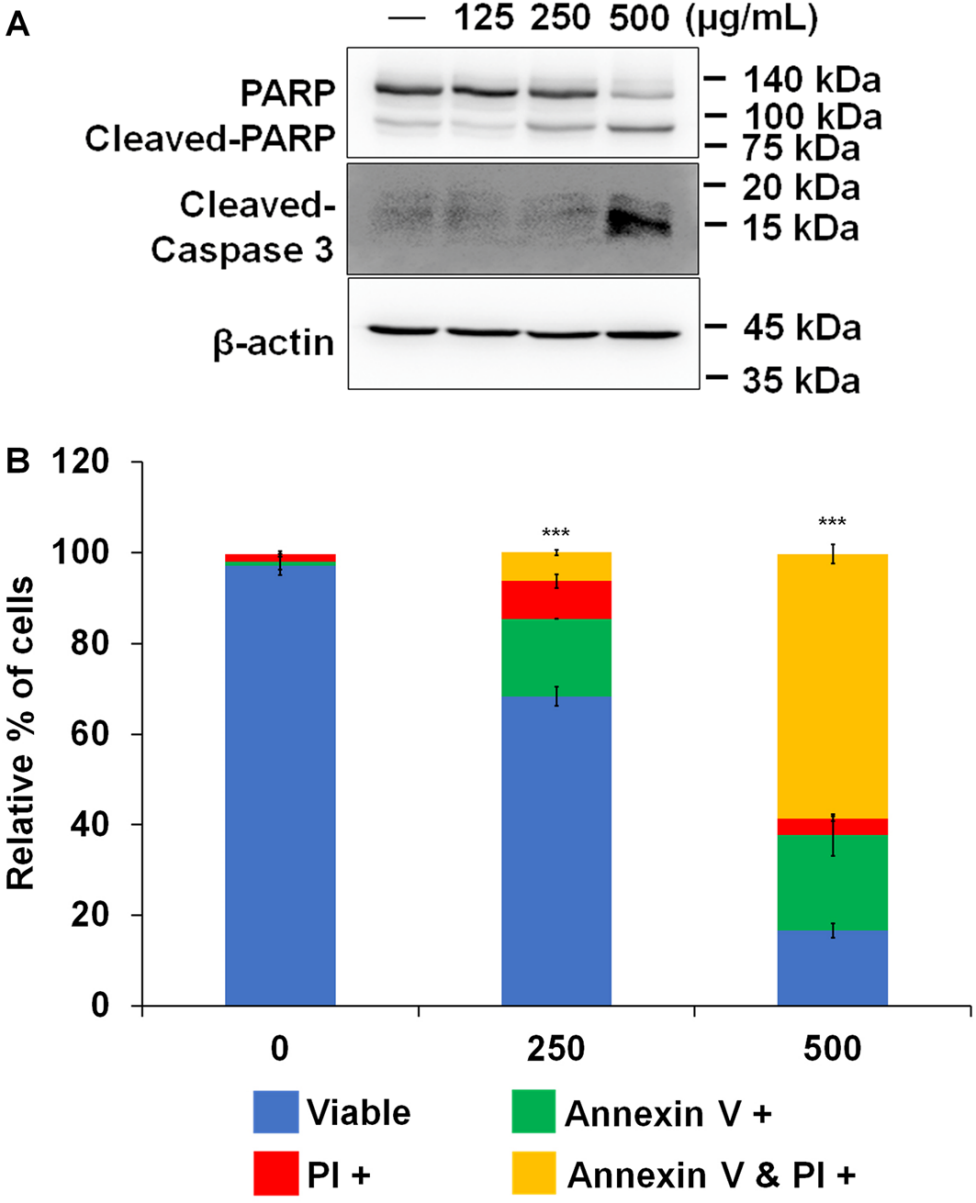
AOFE induces apoptosis in head and neck cancer cells. (**A**) FaDu cells were treated with various concentration of AOFE for 24 h. Western blot analysis was used to analyze protein expression of apoptosis-related factors. (**B**) FaDu cells were treated with indicated concentration of AOFE for 24 h. Apoptotic cell death was analyzed by Arthur image based cytometry after staining with annexin V and PI. The data are shown as the mean ± SD. The Y-axis represents the percent of cells positive for annexin V (green), PI (red), annexin V and PI (yellow), or negative for annexin V and PI (blue). ***p < 0.001 vs. control (comparison of viable cells).

Caspase 3, the major downstream executioner caspase, is cleaved and activated by initiator caspases 8 and 9; then, PARP is cleaved by activated caspase 3, which is a well-characterized apoptotic event^34^. To determine whether AOFE can induce apoptosis in FaDu cells, we analyzed the expression of apoptotic markers by western blotting. As a result, the expression of cleaved PARP and caspase-3 was markedly increased in a dose-dependent manner in AOFE-treated cells (Fig. 2A). Subsequently, AOFE-treated cells stained with annexin V and PI were analyzed using an Arthur image-based cytometer. As a result, annexin-positive and annexin/PI-positive cells were significantly increased in AOFE-treated cells than in UFE-treated cells (Fig. 2B).

### AOFE-induced apoptosis caused by a decrease in IGFBP-2

**Figure 3:**
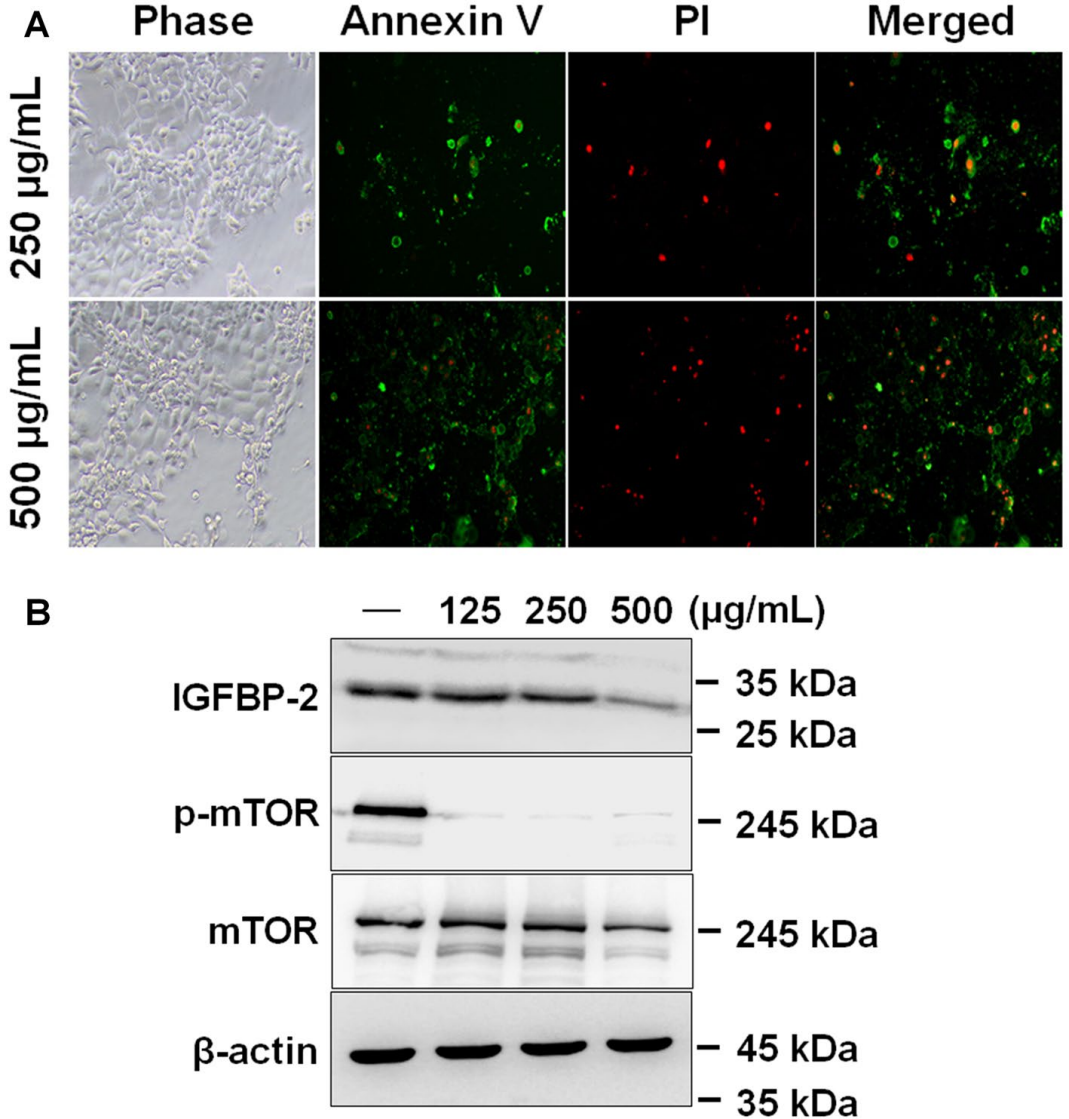
AOFE induces apoptosis through decreased expression of IGFBP-2. (**A**) FaDu cells were treated with indicated concentration of AOFE for 24 h and then stained with annexin V (green) and PI (red). Images of annexin V/PI-stained cells were taken using an Olympus IX71 fluorescence microscope (magnification LCAch N 10X). (**B**) FaDu cells were treated with indicated concentration of AOFE for 24 h. The cell lysates were prepared, and analyzed by western blot analysis using anti-IGFBP-2, anti-phosphor mTOR, anti-mTOR and β-actin antibodies.

Next, to determine whether the abovementioned AOFE-induced decrease in cell viability is associated with apoptotic cell death, AOFE-treated cells were stained with annexin V (green) and PI (red), and then observed using a fluorescence microscope. The number of annexin V/PI-stained AOFE-treated cells increased (Fig. 3A). An apoptosis antibody array was performed to identify the factors associated with AOFE-induced apoptosis. Among the six families of IGFBP, the expression of IGFBP-2 was markedly decreased, but the expression of IGF1, IGF2, and IGF1R remained unchanged in AOFE-treated cells. The expression of caspase-3 was dramatically increased, while TP53 expression was decreased in AOFE-treated cells (Table 1 and Data S1). However, despite the decreased expression of IGFBP-2, no changes were observed in the phosphorylated forms of PTEN (Ser380/382/383) and FAK (Tyr397/861/925) and the non-phosphorylated forms of PTEN (Ser380/382/383) and FAK (Tyr397); however, the non-phosphorylated forms of FAK (Tyr861/925) decreased. Likewise, the phosphorylated forms of PI3K p85 alpha (Tyr607) and subunit alpha/gamma (Tyr467/Tyr199) remained unchanged, while the non-phosphorylated forms decreased in AOFE-treated cells, which was confirmed using Cancer Signaling Phospho Antibody Array (Data S2). Because the mTOR signaling pathway is regulated by IGFBP-2^31^, we confirmed the phosphorylation of mTOR through western blot analysis. When AOFE was treated with various concentrations, the expression of IGFBP-2 was markedly decreased, and the phosphorylation of mTOR was also dramatically decreased in FaDu cells (Fig. 3B).

**Table 1:** Changes in gene expression levels by AOFE treatment in head and neck cancer cells.

Gene symbol	Gene expression levels		
	125 μg/control	250 μg/control	500 μg/control
IGF1	1.014	0.882	1.060
IGF2	0.967	0.926	0.947
IGF1R	1.058	0.980	1.264
IGFBP1	0.866	0.896	0.973
IGFBP2	0.611	0.512	0.688
IGFBP3	1.823	0.874	1.109
IGFBP4	1.015	0.911	1.035
IGFBP5	0.942	0.932	1.011
IGFBP6	1.122	1.079	1.169
TP53	1.231	0.814	0.582
CASP3	1.407	2.378	3.285

Apoptosis antibody array analysis was performed using the Full Moon BioSystems Antibody Microarray.

### AOFE-induced apoptosis inhibited by IGFBP-2

**Figure 4:**
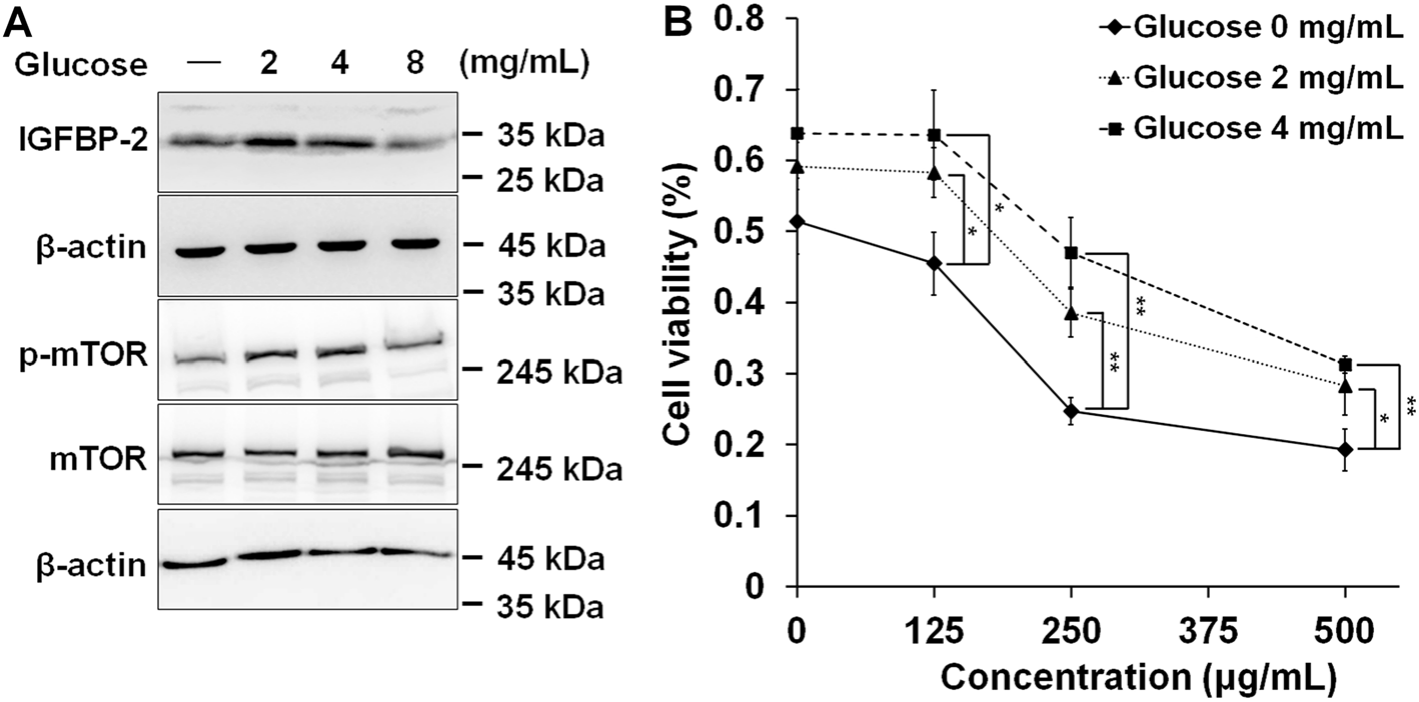
IGFBP-2 can prevent AOFE-induced apoptosis. (**A**) FaDu cells were cultured in a media containing the indicated concentration of glucose for 24 h. The cell lysates were prepared, and analyzed by western blot analysis using anti-IGFBP-2, anti-phosphor mTOR, anti-mTOR and β-actin antibodies. (**B**) After FaDu cells were pretreated with the indicated concentration of glucose for 24 h, AOFE was treated at various concentration. Cell viability was analyzed by MTT assay. The data are presented as the mean ± SD. Statistical analyses were performed using paired t-test. *p < 0.05, **p < 0.01.

Since the expression of IGFBP-2 depends on the glucose concentration in lung adenocarcinoma^30^, the expression of IGFBP-2 in FaDu cells also changes in a glucose dose-dependent manner. Our results showed that the expression of IGFBP-2 increased depending on the glucose concentration, except at a concentration of 8 mg/ml; the phosphorylation of mTOR was also increased (Fig. 4A). In addition, when AOFE was treated with the indicated concentrations after pretreatment with glucose, the cell viability that was reduced by AOFE treatment significantly improved in a glucose dose-dependent manner (Fig. 4B).

### AOFE inhibiting cell viability in other cancer cell lines

**Figure 5:**
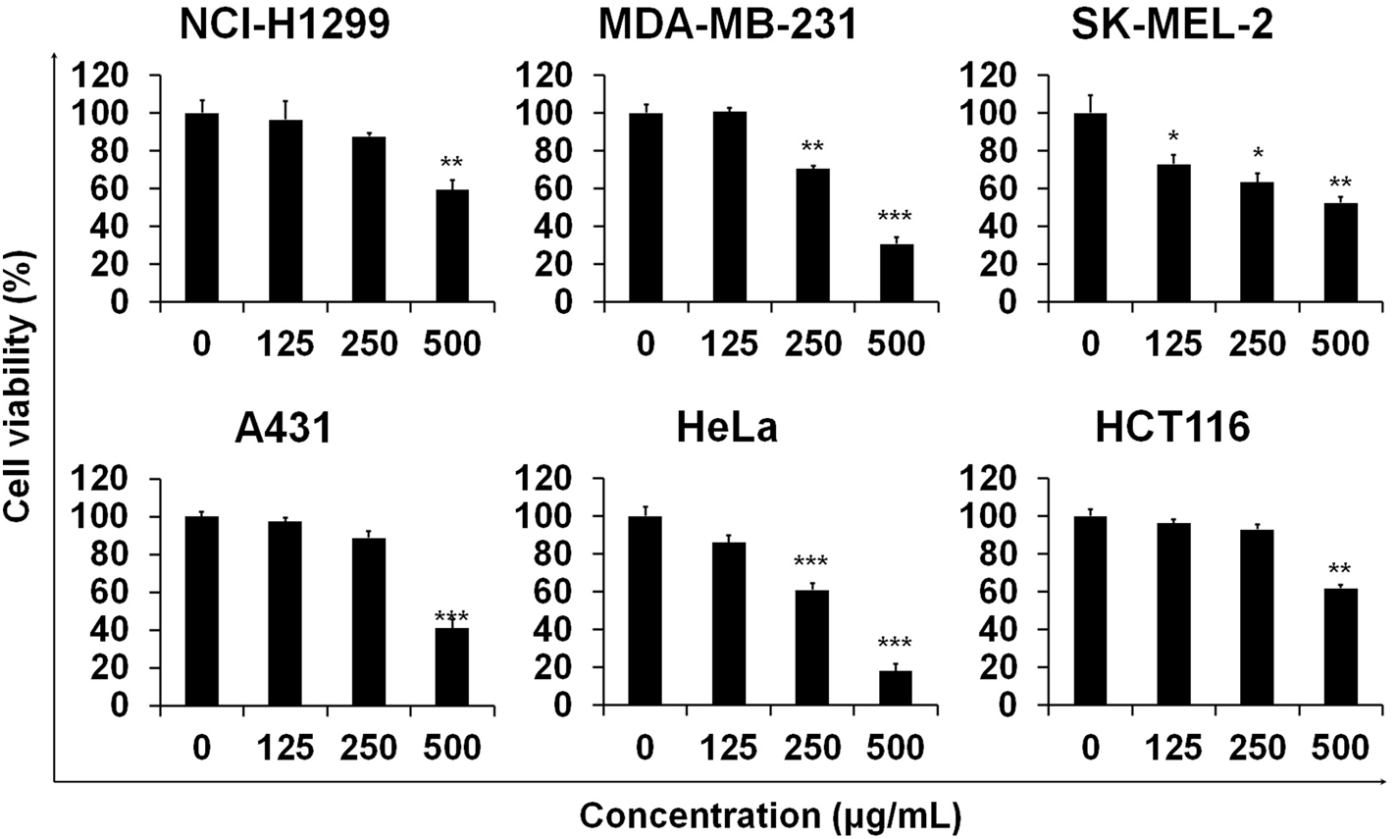
AOFE inhibits cell viability in other cancer cells. NCI-H1299, MDA-MB-231, SK-MEL-2, A431, HeLa, and HCT116 cells were treated with various concentration of AOFE for 24 h. AOFE-induced cytotoxicity was assessed using the MTT assay. The data are presented as the mean ± SD. Statistical analyses were performed using paired t-test. *p < 0.05, **p < 0.01, ***p < 0.001.

To investigate the effects of AOFE on the viability of other cancer cell lines, we performed an MTT assay. Cell viability was significantly decreased at 24 h by AOFE treatment in non-small cell lung carcinoma cell line (NCI-H1299), breast cancer cell line (MDA-MB-231), melanoma cell line (SK-MEL-2), epidermoid carcinoma cell line (A-431), cervical carcinoma cell line (HeLa), and colorectal carcinoma cell line (HCT 116) (Fig. 5).

### Possibility of regulating the expression of IGFBP-2 by gallic acid

**Figure 6:**
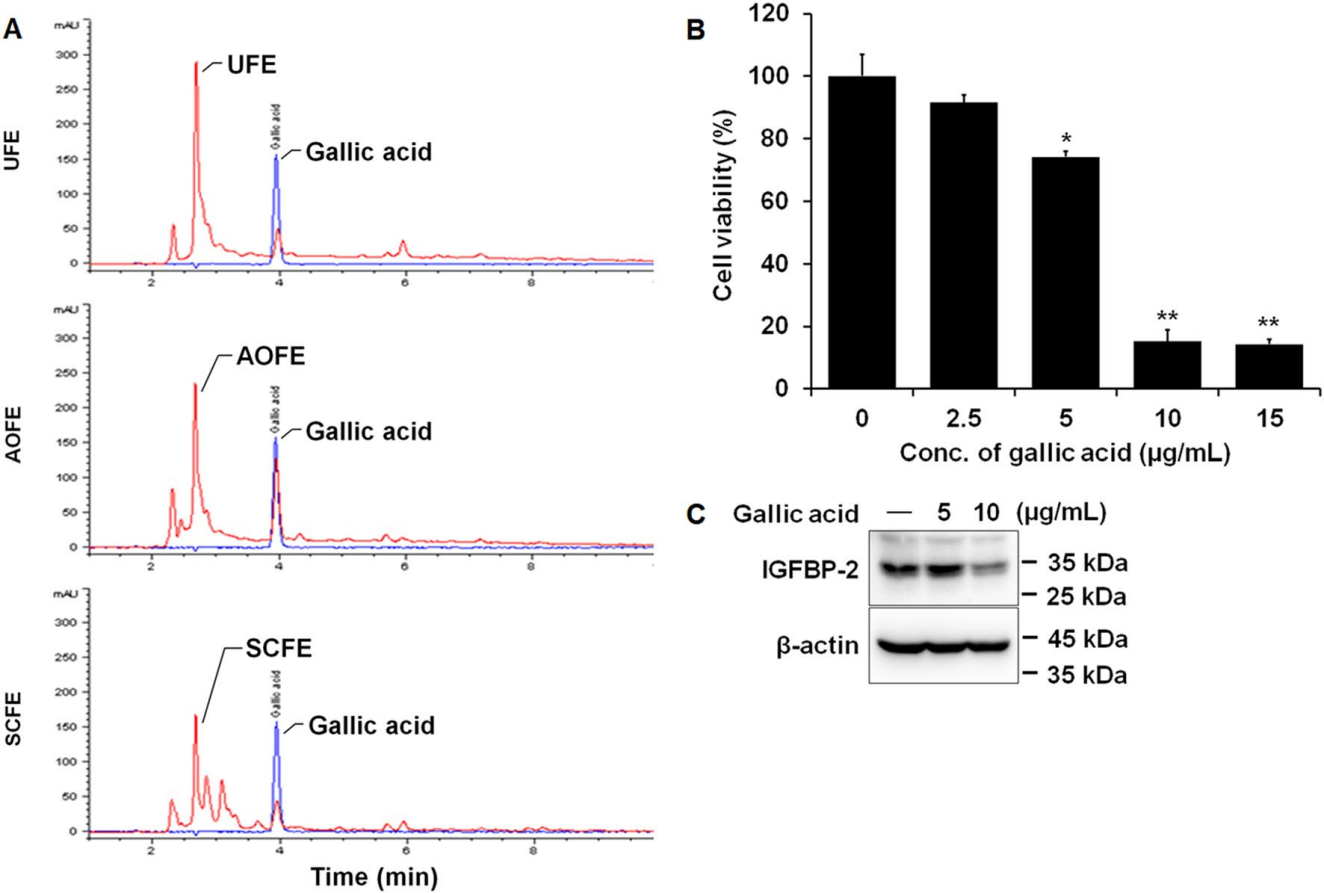
Possibility of regulating the expression of IGFBP-2 by gallic acid with an increase in gallic acid in AOFE. (**A**) The extracts of the CJ dried pericarp were fermented with *A. oryzae* and *S. cerevisiae* was analyzed using HPLC. HPLC chromatograms of unfermented extracts (red line), *A. oryzae* fermented extracts (red line), *S. cerevisiae* fermented extracts (red line) and gallic acid (blue line). The data are presented as the mean ± SD. FaDu cells were treated with the indicated concentration of gallic acid for 24 h. Cell viability was analyzed by MTT assay (**B**), and expression of IGFBP-2 was analyzed by Western blot (**C**). The data are presented as the mean ± SD. Statistical analyses were performed using paired t-test. *p < 0.05, **p < 0.01.

To determine whether the anticancer effect of the water extract, which had no effect, was exhibited by fermentation due to the component change, we performed HPLC and LC/MS/MS. As a result of detecting 14 phenolic compounds using LC/MS/MS, the content of gallic acid was increased by fermentation of *A. oryzae* (Table 2), and HPLC also demonstrated that gallic acid increased in fermented extract (11.37 ± 3.18 μg/mg) compared to the unfermented extract (35.77 ± 5.54 μg/mg) (*P* < 0.01) (Fig. 6A). To determine whether gallic acid affects cell viability and expression of IGFBP-2 in FaDu cells, MTT assay and Western blot analysis were performed. As a result, cell viability was significantly reduced by gallic acid (Fig. 6B), and the expression of IGFBP-2 was also reduced (Fig. 6C).

**Table 2:** Contents of quantified phenolic compounds contained in AOFE.

	Concentration (μg/mg)		
	UFE	AOFE	SCFE
4-Hydroxybenzoic acid	0.014 ± 0.0007	0.022 ± 0.0009^a^	0.71 ± 0.0075^b^
Caffeic acid	ND	ND	ND
Syringic acid	0.020 ± 0.0004	ND	ND
Vanillic acid	0.013 ± 0.0004	ND	ND
Coumaric acid	0.041 ± 0.0035	ND	0.016 ± 0.0016^b^
Rutin	0.015 ± 0.0027	ND	ND
Ferulic acid	0.023 ± 0.0018	ND	ND
Oxyresveratrol	ND	ND	ND
Naringein	ND	0.001 ± 0.0000	ND
Resveratol	ND	ND	ND
Benzoic acid	ND	ND	ND
Nicotinic acid	0.003 ± 0.0007	0.007 ± 0.0026	0.067 ± 0.0095^b^
Gallic acid	1.620 ± 0.0730	5.596 ± 0.1746^a^	1.787 ± 0.0765
Protocatechuic acid	0.761 ± 0.0432	0.643 ± 0.0220^a^	0.375 ± 0.0124^b^

Different superscript letters indicate significant differences in mean values at p < 0.05 between unfermented extracts and fermented extracts. Results are expressed as mean ± SD of three sample replicates.

UFE, unfermented extracts; AOFE, *A. oryzae* fermented extracts; SCFE, *S. cerevisiae* extracts. ND, Not detected.

## Discussion

Methanol extract from dried fruit of CJ showed a growth inhibitory effect in leukemia^22^. In this study, the ethanol extract from dried pericarp of CJ also reduced the cell viability, but showed strong cytotoxicity in normal cells. The bioactive components obtained from plants have improved biological activities through fermentation^35–37^. The anticancer effect of *A. oryzae* extracts fermented at 30 °C or 35 °C was remarkably improved, while the water extract of dried CJ pericarp showed no anticancer activity. Flow cytometry analysis using annexin V and PI revealed that the change in cell viability reduced by AOFE treatment was due to apoptosis. In addition, apoptotic factors at the molecular level were investigated using western blot analysis and showed that apoptosis was related to the caspase-dependent process.

Membrane-associated IGFBP-2 promotes cell proliferation, survival, migration, and invasion, and is closely related to the prevention of apoptosis in various types of cancers^28–30,38^. Intracellular IGFBP-2 exhibits antiapoptotic properties, which proved that overexpression of IGFBP-2 reduces the expression of caspase-3 in lung adenocarcinoma. In other words, the effect of intracellular IGFBP-2 is opposite to that of caspase-3^30^. Our results also showed that caspase-3 expression significantly increased when IGFBP-2 expression was decreased by AOFE treatment through an apoptosis antibody array. FAK is phosphorylated by IGFBP-2, which enhances cell viability^39^, and FAK phosphorylates PI3K^40^. Furthermore, IGFBP-2 regulates the PI3K/AKT/mTOR signaling pathway and is inversely correlated with PTEN^41^. Our results showed that despite the reduction of IGFBP-2 by AOFE treatment, it had no effect on the phosphorylated and non-phosphorylated forms of PTEN (Ser380/382/383) and phosphorylated form of FAK (Tyr397/861/925). However, the levels of the non-phosphorylated form of FAK (Tyr861/925) decreased. In addition, the phosphorylated form of PI3K p85 alpha (Tyr607) and subunit alpha/gamma (Tyr467/Tyr199) remained unchanged, but the non-phosphorylated form was reduced. Despite these results, AOFE could regulate the mTOR signaling pathway, after which changes in mTOR phosphorylation were confirmed.

When knocked down through the siRNA of IGFBP-2, cleaved PARP is increased and the expression of IGFBP-2 is also increased depending on the concentration of glucose^30^. PARP cleavage was increased by AOFE treatment, and the expression of IGFBP-2 and mTOR phosphorylation increased depending on the glucose concentration. These results are consistent with those of previous studies, which reported that the mTOR signaling pathway is regulated by IGFBP-2^31^, and show that AOFE can regulate the IGFBP-2/mTOR signaling pathway.

AOFE have anticancer effects in lung cancer, breast cancer, skin cancer, cervical cancer, and colon cancer cells. Increase in the expression of IGFBP-2 in lung cancer cells^39^, breast cancer cells^42^, and cervical cancer cells^43^ increased the proliferation of cells. Conversely, decrease in the expression of IGFBP-2 in skin cancer cells^44^ and colorectal cancer cells^45^ decreased the proliferation of cells. In these cancer cells, IGFBP-2 acts as an oncogene, and the decrease in cell viability by AOFE treatment may be attributed to the possibility that the expression of IGFBP-2 may be regulated by AOFE treatment.

Moreover, the content of gallic acid was markedly increased by fermentation of *A. Oryzae*. Gallic acid has been reported to have anti-cancer effects in human hepatocellular carcinoma, human small cell lung cancer, cervical cancer, and breast cancer^46–49^. Since gallic acid and caffeic acid are abundant in pineapple vinegar^50^, and it has been reported that pineapple vinegar reduces the angiogenesis-related gene IGFBP-2 in mammary gland cells^51^, this suggests that gallic acid may be associated with IGFBP-2 expression, and our results showed that gallic acid may regulate IGFBP-2.

Taken together, IGFBP-2 is positively associated with tumor growth and progression^52^, and AOFE exhibits anticancer effects by reducing the expression of IGFBP-2. Therefore, AOFE appears to be a promising candidate for the treatment of head and neck cancer.

## Methods

### Extraction and fermentation

Fruits of CJ were collected from the arboretum located in Wando of Jeollanam-do Forest Resources Research Institute (126° 6680.07″ E longitude and 34° 3464.90″ N latitude) according to the Wild Animal and Plant Protection Act of Korea. The morphological identification of the specimen was performed at Ajou University (Suwon, Gyeonggi-do, Korea) and registered in the Korea Biodiversity Information System (Specimen number: AJHA200102151044). Experimental research on plants were carried out in compliance with relevant institutional, national, and international guidelines and legislation. After washing and drying the fruits of CJ, pericarps were separated and extracted with water and ethanol using an ASE350 accelerated solvent extractor (Dionex, Sunnyvale, CA, USA). After centrifuging the extracts, the supernatants were filtered using a 0.45-μm syringe filter and then lyophilized using a freeze dryer. The dried pericarps of the CJ were ground into powder using a blender. 2 g of the ground powder was mixed with 40 mL of distilled water in a 250-mL Erlenmeyer flask, sterilized at 121 °C for 15 min, and then cooled below 30 °C. 0.2% (0.08 g) of *Aspergillus oryzae* (Chungmoo Fermentation Co., Ltd., Ulsan, Republic of Korea) and 1% (1 g) of *Saccharomyces cerevisiae* (UAB Ekoproduktas, Panevezys, Lithuania) powder were added to each Erlenmeyer flask and then cultivated in a shaking incubator at 120 rpm at 30 °C or 35 °C for 3 days. Fermented products were transferred into a 50 mL conical centrifuge tube and centrifuged at 2600×*g* for 20 min. The supernatants were filtered and transferred into a new 50 mL conical centrifuge tube and stored at − 72 °C. After 24 h, the fermented extracts of *A. oryzae* (AOFE) and *S. cerevisiae* (SCFE) were lyophilized using a freeze dryer.

### Cell culture

All cell lines were purchased from the Korean Cell Line Bank (Seoul, Republic of Korea). The human hypopharyngeal carcinoma cell line FaDu, human epidermoid carcinoma cell line A431, and human cervical carcinoma cell line HeLa were cultured in Minimum Essential Medium (Gibco, Grand Island, NY, USA). The human non-small cell lung carcinoma cell line NCI-H1299, human breast cancer cell line MDA-MB-231, human melanoma cell line SK-MEL-2, and human colorectal carcinoma cell line HCT 116 were cultured in RPMI-1640 Medium (Gibco, Grand Island, NY, USA). All media were supplemented with 10% heat-inactivated fetal bovine serum, 100 U/mL penicillin, and 100 μg/mL streptomycin (Gibco, Grand Island, NY, USA).

### MTT assay

The cells were plated in 96-well plates at a density of 20,000 cells per well. After incubating overnight, the cells were treated with UFE, AOFE, and SCFE using various concentrations. After 24 h, the culture medium was replaced with fresh medium, 20 μL of a 5 mg/mL stock solution of 3-(4,5-dimetylthiazol-2-yl)-2,5-diphenyltetrazolium bromide (Sigma Aldrich) was added to each well, and the cells were incubated for 4 h at 37 °C. The supernatant was removed, and 100 μL of DMSO was added to each well. The plates were incubated on a horizontal shaker for 30 min at room temperature, and the optical density was measured at an absorbance of 570 nm using a Biochrom Asys UVM 340 microplate reader (Cambridge, UK).

### Western blot analysis

The cells were plated in 100-mm dishes at a density of 1,600,000 cells. After incubating overnight, the cells were treated with AOFE using the indicated concentrations. After incubating for 24 h, the cells were washed with PBS and lysed in RIPA buffer (Biosolution Co., Ltd., Seoul, Republic of Korea) containing protease inhibitor cocktail III (ProGEN, Gyeonggi-do, Republic of Korea). The cell lysates were centrifuged at 16,000×*g* for 20 min at 4 °C, and the supernatant was used for western blot analysis. The protein concentrations were quantified using a Pierce Rapid Gold BCA Protein Assay Kit (Thermo Fisher, Waltham, MA, USA). The proteins (50 μg) were separated by sodium dodecyl-sulfate polyacrylamide gel electrophoresis and transferred to PVDF membranes (Millipore, Bedford, USA). Membranes were blocked with TBST containing 5% skim milk at room temperature for 1 h and then incubated overnight at 4 °C with primary antibodies against PARP (#9542, 1:1000), cleaved caspase-3 (#9664, 1:1000), IGFBP-2 (#3922, 1:1000), mTOR (#2983, 1:1000), phospho-mTOR (#5536, 1:1000), and β-actin (#3700, 1:5000). Membranes were washed four times with TBST buffer and incubated with a secondary anti-mouse HRP (#7076, 1:5000) and anti-rabbit HRP antibody (#7074, 1:5000). All antibodies were purchased from Cell Signaling Technology. Amersham ECL western blotting detection reagent (RPN2106, GE healthcare, Buckinghamshire, UK) was used for detection, and band images were acquired using the Fusion Solo imaging system (Fusion SOLO, Vilber Lourmat, France).

### Fluorescence microscopy

The cells were seeded in 24-well plates at a density of 160,000 cells per well. After incubating overnight, the cells were treated with AOFE using the indicated concentrations. After 24 h, the cells were washed with PBS and incubated with 5 μL Alexa Fluor 488 annexin V and 1 μL 100 μg/mL PI working solution (Molecular Probes, OR, USA) for 15 min at room temperature. The cells were washed with PBS and fixed with 4% paraformaldehyde for 10 min. The cell images were photographed using a fluorescence microscope (Olympus IX71, Tokyo, Japan).

### Arthur image-based cytometric assay

The cells were seeded in 100-mm dishes at a density of 1,600,000 cells. After incubating overnight, the cells were treated with AOFE using the indicated concentrations. After 24 h, the cells were washed with PBS and treated with trypsin–EDTA solution. After harvesting, the cells were washed with cold PBS and resuspended in 1× annexin-binding buffer. Each 100 μL of cell suspension was incubated with 5 μL Alexa Fluor 488 annexin V and 1 μL 100 μg/mL PI working solution (Molecular Probes, OR, USA) for 15 min at room temperature. The prepared samples were analyzed using an Arthur image-based cytometer (NanoEnTek Inc., Seoul, Republic of Korea).

### Antibody microarray

Cells were plated in 100-mm dishes at a density of 1,600,000 cells. After incubating overnight, the cells were treated with AOFE using the indicated concentrations. After incubating for 24 h, the cells were harvested by scraping and washed with PBS. Apoptosis Antibody Array and Cancer Signaling Phospho Antibody Array were designed and manufactured by Full Moon Biosystems, Inc. (Sunnyvale, CA). Antibody array and analysis were performed using the customized service provided by Ebiogen Inc. (Seoul, Republic of Korea).

### Liquid chromatography-tandem mass spectrometery

In order to quantitatively analyze the polyphenolic compounds contained in AOFE, LC/MS/MS analysis was performed on 15 representative polyphenols. Standard substances used for quantitative analysis are 4-hydroxy benzoic acid, caffeic acid, syringic acid, vanillic acid, coumaric acid, rutin, ferulic acid, oxyresveratrol, naringenin, resveratrol, benzoic acid, nicotinic acid, gallic acid and protocatechuic acid. LC/MS/MS (AB SCIEX 4000 Q Trap LC/MS/MS System, Shimadzu LC 20A System, Japan) was used for quantitative analysis of the analyte. Chromatographic separtation was achieved on a column of Gemini C18 (50 mm*2.0 mm, 3 μm) with a gradient mode of mobile phase. The mobile phase were 0.1% formic acid solution (A) and acetonitrile (B) containing 0.1% formic acid. The gradient mode of mobile phase was as follows; liquid B was started at 0%, increased to 20% in 0.5 min, increased to 80% in 2 min, maintained until 2.5 min, then lowered back to 20% until 2.6 min and maintained for 6 min. The column oven temperature was 40 ℃ and the temperature of the autosampler was 15 ℃. The flow rate was 0.3 mL/min, and the sample injection volume was 10 μL. The detection for quantitative analysis was accomplished by a tandem mass spectrometer in MRM (multiple reaction monitoring) mode. MS/MS conditions were Turbo Ion Spray method, detector temperature was 400 ℃, spray voltage was − 4500 V in negative mode, curtain gas (N2 gas) was 30 psi and Gas 1 and Gas 2 were both 50 psi.

### High-performance liquid chromatography

Agilent 1200 series HPLC (Agilent Technologies, CA, USA) was hired to qualitatively analyze the polyphenols contained in the extracts. Standard substance selected for representative polyphenols are gallic acid. YMC-Pack ODS-AM (250 × 4.6 mm ID 5 µm, 12 nm, YMC CO., Japan) was used as the column, and the mobile phase were 0.1% acetic acid in water (solvent A) and methanol (solvent B). The column oven temperature was 40 °C, the wavelength of the UV detector was 254 nm, and the flow rate was 1.0 mL/min.

### Statistical analysis

All data were presented as mean ± SD. Statistical analyses were performed using the paired t-test. Between-group differences with a p value of < 0.05 were considered significant. Statistical analyses were performed using IBM SPSS Statistics version 20.

## Supplementary Information

Supplementary Figure S1.

Supplementary Information 1.

Supplementary Information 2.

## References

[1] Shin SA, Moon SY, Kim WY, Paek SM, Park HH, Lee CS (2018). Structure-based classification and anti-cancer effects of plant metabolites. Int. J. Mol. Sci..

[2] Gali-Muhtasib H, Hmadi R, Kareh M, Tohme R, Darwiche N (2015). Cell death mechanisms of plant-derived anticancer drugs: Beyond apoptosis. Apoptosis.

[3] Holohan C, Van Schaeybroeck S, Longley DB, Johnston PG (2013). Cancer drug resistance: An evolving paradigm. Nat. Rev. Cancer..

[4] Longley DB, Johnston PG (2005). Molecular mechanisms of drug resistance. J. Pathol..

[5] van Leeuwen IM, Rao B, Sachweh MC, Laín S (2012). An evaluation of small-molecule p53 activators as chemoprotectants ameliorating adverse effects of anticancer drugs in normal cells. Cell Cycle.

[6] Fridlender M, Kapulnik Y, Koltai H (2015). Plant derived substances with anti-cancer activity: From folklore to practice. Front. Plant. Sci..

[7] Fernald K, Kurokawa M (2013). Evading apoptosis in cancer. Trends Cell Biol..

[8] Madden EC, Gorman AM, Logue SE, Samali A (2020). Tumour cell secretome in chemoresistance and tumour recurrence. Trends cancer..

[9] Schmidt BM, Ribnicky DM, Lipsky PE, Raskin I (2007). Revisiting the ancient concept of botanical therapeutics. Nat. Chem. Biol..

[10] Gordaliza M (2007). Natural products as leads to anticancer drugs. Clin. Transl. Oncol..

[11] Ijaz S, Akhtar N, Khan MS, Hameed A, Irfan M, Arshad MA, Ali S, Asrar M (2018). Plant derived anticancer agents: A green approach towards skin cancers. Biomed. Pharmacother..

[12] Valli M, Pivatto M, Danuello A, Castro-Gamboa I, Silva DHS, Cavalheiro AJ, Araújo ÂR, Furlan M, Lopes MN, Bolzani VDS (2012). Tropical biodiversity: Has it been a potential source of secondary metabolites useful for medicinal chemistry?. Quim. Nova..

[13] Piao MJ, Yoo ES, Koh YS, Kang HK, Kim J, Kim YJ, Kang HH, Hyun JW (2011). Antioxidant effects of the ethanol extract from flower of *Camellia japonica* via scavenging of reactive oxygen species and induction of antioxidant enzymes. Int. J. Mol. Sci..

[14] Kim S, Jung E, Shin S, Kim M, Kim YS, Lee J, Park D (2012). Anti-inflammatory activity of *Camellia japonica* oil. BMB Rep..

[15] Lee JH, Kim JW, Ko NY, Mun SH, Kim DK, Kim JD, Kim HS, Lee KR, Kim YK, Radinger M, Her E, Choi WS (2008). *Camellia japonica* suppresses immunoglobulin E-mediated allergic response by the inhibition of Syk kinase activation in mast cells. Clin. Exp. Allergy..

[16] Kim KY, Davidson PM, Chung HJ (2001). Antibacterial activity in extracts of *Camellia japonica* L. petals and its application to a model food system. J. Food Prot..

[17] Miura D, Kida Y, Nojima H (2007). Camellia oil and its distillate fractions effectively inhibit the spontaneous metastasis of mouse melanoma BL6 cells. FEBS Lett..

[18] Jung E, Lee J, Baek J, Jung K, Lee J, Huh S, Kim S, Koh J, Park D (2007). Effect of *Camellia japonica* oil on human type I procollagen production and skin barrier function. J. Ethnopharmacol..

[19] Lee H-H, Paudel KR, Jeong J, Wi A-J, Park W-S, Kim D-W, Oak M-H (2016). Antiatherogenic effect of *Camellia japonica* fruit extract in high fat diet–fed rats. Based Complement. Alternat. Med..

[20] Park JC, Hur JM, Park JG, Hatano T, Yoshida T, Miyashiro H, Min BS, Hattori M (2002). Inhibitory effects of Korean medicinal plants and camelliatannin H from *Camellia japonica* on human immunodeficiency virus type 1 protease. Phytother. Res..

[21] Akihisa T, Tokuda H, Ukiya M, Suzuki T, Enjo F, Koike K, Nikaido T, Nishino H (2004). 3-epicabraleahydroxylactone and other triterpenoids from camellia oil and their inhibitory effects on Epstein–Barr virus activation. Chem. Pharm. Bull..

[22] Kuete V, Seo EJ, Krusche B, Oswald M, Wiench B, Schröder S, Greten HJ, Lee IS, Efferth T (2013). Cytotoxicity and pharmacogenomics of medicinal plants from traditional Korean medicine. Evid. Based Complement. Alternat. Med..

[23] Park SH, Shim BS, Yoon JS, Lee HH, Lee HW, Yoo SB, Wi AJ, Park WS, Kim HJ, Kim DW, Oak MH (2015). Vascular protective effect of an ethanol extract of *Camellia japonica* fruit: Endothelium-dependent relaxation of coronary artery and reduction of smooth muscle cell migration. Oxid. Med. Cell Longev..

[24] Akanda MR, Park BY (2017). Involvement of MAPK/NF-κB signal transduction pathways: *Camellia japonica* mitigates inflammation and gastric ulcer. Biomed. Pharmacother..

[25] Jeon H, Kim JY, Choi JK, Han E, Song CL, Lee J, Cho YS (2018). Effects of the Extracts from Fruit and Stem of Camellia japonica on Induced Pluripotency and Wound Healing. J. Clin. Med..

[26] Höflich A, Lahm H, Blum W, Kolb H, Wolf E (1998). Insulin-like growth factor-binding protein-2 inhibits proliferation of human embryonic kidney fibroblasts and of IGF-responsive colon carcinoma cell lines. FEBS Lett..

[27] Reeve JG, Morgan J, Schwander J, Bleehen NM (1993). Role for membrane and secreted insulin-like growth factor-binding protein-2 in the regulation of insulin-like growth factor action in lung tumors. Cancer Res..

[28] Zhu H, Zhang Y, Geng Y, Lu W, Yin J, Li Z, Huang L, Liu H, Xu N (2019). IGFBP2 promotes the EMT of colorectal cancer cells by regulating E-cadherin expression. Int. J. Clin. Exp. Pathol..

[29] Han S, Li Z, Master LM, Master ZW, Wu A (2014). Exogenous IGFBP-2 promotes proliferation, invasion, and chemoresistance to temozolomide in glioma cells via the integrin β1-ERK pathway. Br. J. Cancer..

[30] Migita T, Narita T, Asaka R, Miyagi E, Nagano H, Nomura K, Matsuura M, Satoh Y, Okumura S, Nakagawa K, Seimiya H, Ishikawa Y (2010). Role of insulin-like growth factor binding protein 2 in lung adenocarcinoma: IGF-independent antiapoptotic effect via caspase-3. Am. J. Pathol..

[31] Zeng L, Perks CM, Holly JM (2015). IGFBP-2/PTEN: A critical interaction for tumours and for general physiology?. Growth Horm. IGF Res..

[32] Dean SJ, Perks CM, Holly JM, Bhoo-Pathy N, Looi LM, Mohammed NA, Mun KS, Teo SH, Koobotse MO, Yip CH, Rhodes A (2014). Loss of PTEN expression is associated with IGFBP2 expression, younger age, and late stage in triple-negative breast cancer. Am. J. Clin. Pathol..

[33] Matuschek G, Rudoy M, Peiper M, Gerber PA, Hoff NP, Buhren BA, Flehmig B, Budach W, Knoefel WT, Bojar H, Prisack HB, Steinbach G, Shukla V, Schwarz A, Kammers K, Erhardt A, Scherer A, Bölke E, Schauer M (2011). Do insulin-like growth factor associated proteins qualify as a tumor marker? Results of a prospective study in 163 cancer patients. Eur. J. Med. Res..

[34] Wen X, Lin ZQ, Liu B, Wei YQ (2012). Caspase-mediated programmed cell death pathways as potential therapeutic targets in cancer. Cell Prolif..

[35] Villarreal-Soto SA, Beaufort S, Bouajila J, Souchard J-P, Renard T, Rollan S, Taillandier P (2019). Impact of fermentation conditions on the production of bioactive compounds with anticancer, anti-inflammatory and antioxidant properties in Kombucha tea extracts. Process Biochem..

[36] Yu Y, Zhang J, Wang J, Sun B (2019). The anti-cancer activity and potential clinical application of rice bran extracts and fermentation products. RSC Adv..

[37] Kim YS, Kim EK, Tang Y, Hwang JW, Natarajan SB, Kim WS, Moon SH, Jeon BT, Park PJ (2016). Antioxidant and anticancer effects of extracts from fermented *Haliotis discus* hannai with *Cordyceps militaris* mycelia. Food Sci. Biotechnol..

[38] Lu H, Wang L, Gao W, Meng J, Dai B, Wu S, Minna J, Roth JA, Hofstetter WL, Swisher SG, Fang B (2013). IGFBP2/FAK pathway is causally associated with dasatinib resistance in non-small cell lung cancer cells. Mol. Cancer. Ther..

[39] Xia H, Nho RS, Kahm J, Kleidon J, Henke CA (2004). Focal adhesion kinase is upstream of phosphatidylinositol 3-kinase/Akt in regulating fibroblast survival in response to contraction of type I collagen matrices via a beta 1 integrin viability signaling pathway. J. Biol. Chem..

[40] Zeng ZZ, Jia Y, Hahn NJ, Markwart SM, Rockwood KF, Livant DL (2006). Role of focal adhesion kinase and phosphatidylinositol 3′-kinase in integrin fibronectin receptor-mediated, matrix metalloproteinase-1-dependent invasion by metastatic prostate cancer cells. Cancer Res..

[41] Mehrian-Shai R, Chen CD, Shi T, Horvath S, Nelson SF, Reichardt JK, Sawyers CL (2007). Insulin growth factor-binding protein 2 is a candidate biomarker for PTEN status and PI3K/Akt pathway activation in glioblastoma and prostate cancer. Proc. Natl. Acad. Sci. USA.

[42] Du Y, Wang P (2019). Upregulation of MIIP regulates human breast cancer proliferation, invasion and migration by mediated by IGFBP2. Pathol. Res. Pract..

[43] Dai N, Ji F, Wright J, Minichiello L, Sadreyev R, Avruch J (2017). IGF2 mRNA binding protein-2 is a tumor promoter that drives cancer proliferation through its client mRNAs IGF2 and HMGA1. Elife.

[44] Zhao S, Wu L, Kuang Y, Su J, Luo Z, Wang Y, Li J, Zhang J, Chen W, Li F, He Y, Tao J, Zhou J, Xu X, Peng C, Chen X (2018). Downregulation of CD147 induces malignant melanoma cell apoptosis via the regulation of IGFBP2 expression. Int. J. Oncol..

[45] Ben-Shmuel A, Shvab A, Gavert N, Brabletz T, Ben-Ze'ev A (2013). Global analysis of L1-transcriptomes identified IGFBP-2 as a target of ezrin and NF-κB signaling that promotes colon cancer progression. Oncogene.

[46] Sun G, Zhang S, Xie Y, Zhang Z, Zhao W (2016). Gallic acid as a selective anticancer agent that induces apoptosis in SMMC-7721 human hepatocellular carcinoma cells. Oncol. Lett..

[47] Wang R, Ma L, Weng D, Yao J, Liu X, Jin F (2016). Gallic acid induces apoptosis and enhances the anticancer effects of cisplatin in human small cell lung cancer H446 cell line via the ROS-dependent mitochondrial apoptotic pathway. Oncol. Rep..

[48] Aborehab NM, Osama N (2019). Effect of Gallic acid in potentiating chemotherapeutic effect of Paclitaxel in HeLa cervical cancer cells. Cancer Cell Int..

[49] Wang K, Zhu X, Zhang K, Zhu L, Zhou F (2014). Investigation of gallic acid induced anticancer effect in human breast carcinoma MCF-7 cells. J. Biochem. Mol. Toxicol..

[50] Mohamad NE, Yeap SK, Lim KL, Mohd Yusof H, Beh BK, Tan SW, Ho WY, Sharifuddin SA, Jamaluddin A, Long K, Rahman NMANA, Alitheen NB (2015). Antioxidant effects of pineapple vinegar in reversing of paracetamol-induced liver damage in mice. Chin. Med..

[51] Mohamad NE, Abu N, Yeap SK, Lim KL, Romli MF, Sharifuddin SA, Long K, Alitheen NB (2019). Apoptosis and metastasis inhibitory potential of pineapple vinegar against mouse mammary gland cells in vitro and in vivo. Nutr. Metab..

[52] Hoeflich A, Reisinger R, Lahm H, Kiess W, Blum WF, Kolb HJ, Weber MM, Wolf E (2001). Insulin-like growth factor-binding protein 2 in tumorigenesis: protector or promoter?. Cancer Res..

