# Supplementary material for: Anticancer properties of dried-pericarp water extracts of Camellia japonica L. fermented with Aspergillus oryzae through regulation of IGFBP-2/mTOR pathway
Source: Sci Rep. 2021 Nov 2;11:21527. doi: 10.1038/s41598-021-01127-3 (PMC8564518; doi:10.1038/s41598-021-01127-3)
Supplement: Supplementary file 1 — Supplementary Figure S1. [file 41598_2021_1127_MOESM1_ESM.pdf]

Anticancer properties of dried-pericarp water extracts of *Camellia japonica* L. fermented with *Aspergillus oryzae* through regulation of IGFBP-2/mTOR pathway

Eugene Cho[1], Jin Kim[2], Da Hye Jeong[1] and Hyoun Woo Kim[1],*

[1]Jeollanam-do Forest Resource Research Institute, Naju, Jeonnam 58213, Republic of Korea

[2]Gwangju health university, Gwangsan-gu, Gwangju, 62287, Republic of Korea

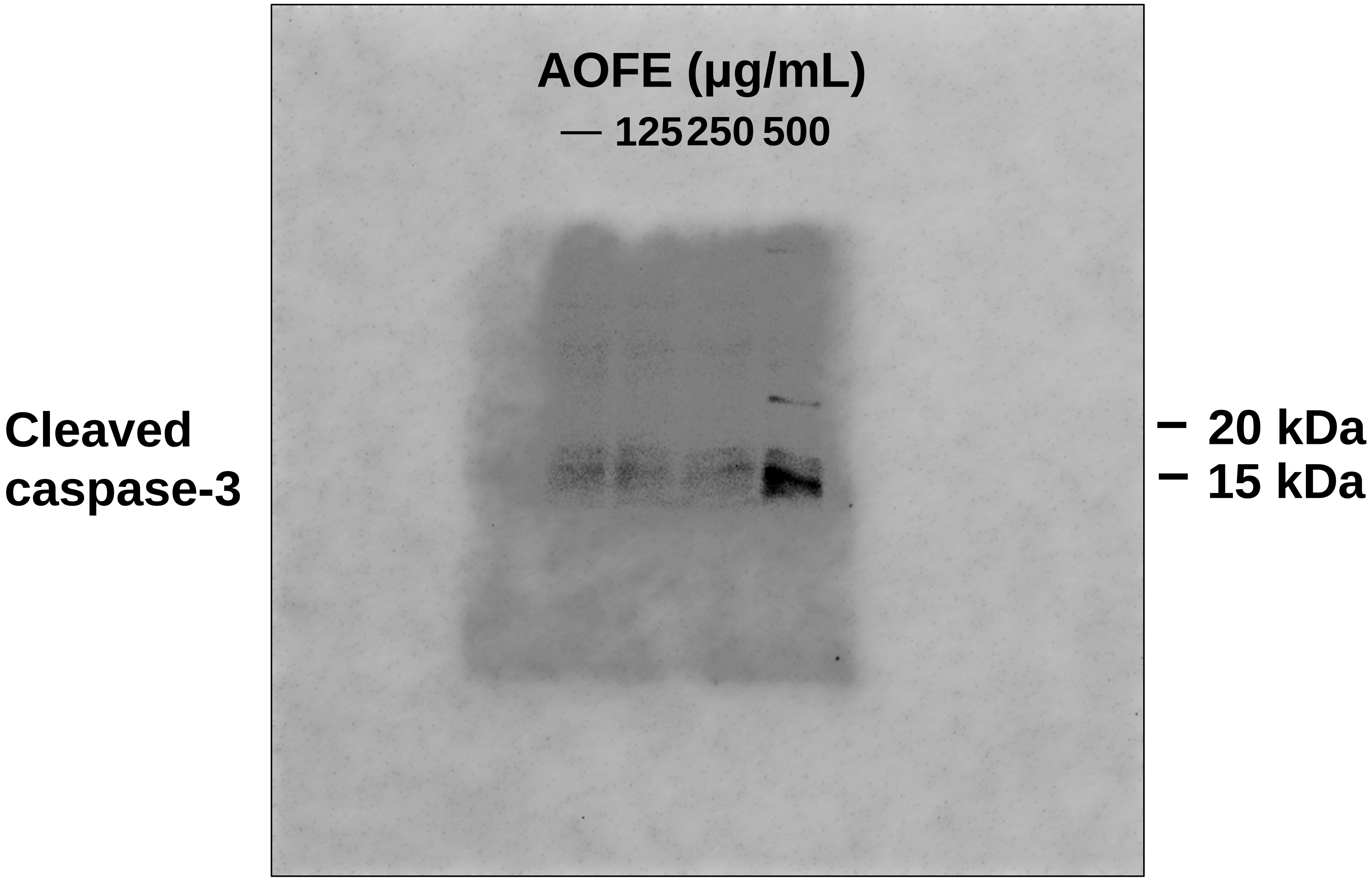

Supplementray Figure S1. Uncropped image of cleaved caspase-3 in figure 2A

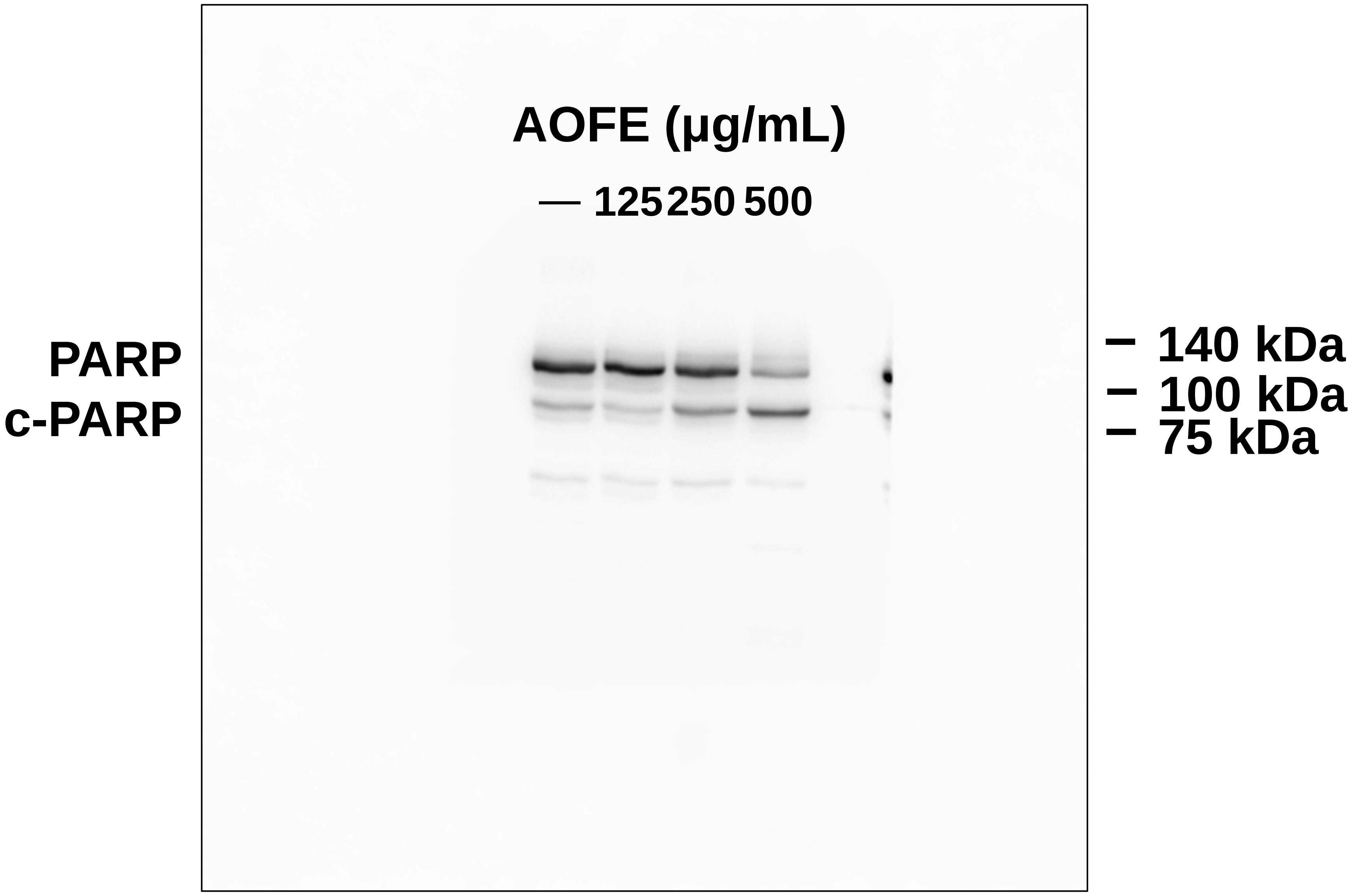

Supplementray Figure S1. Uncropped image of PARP in figure 2A

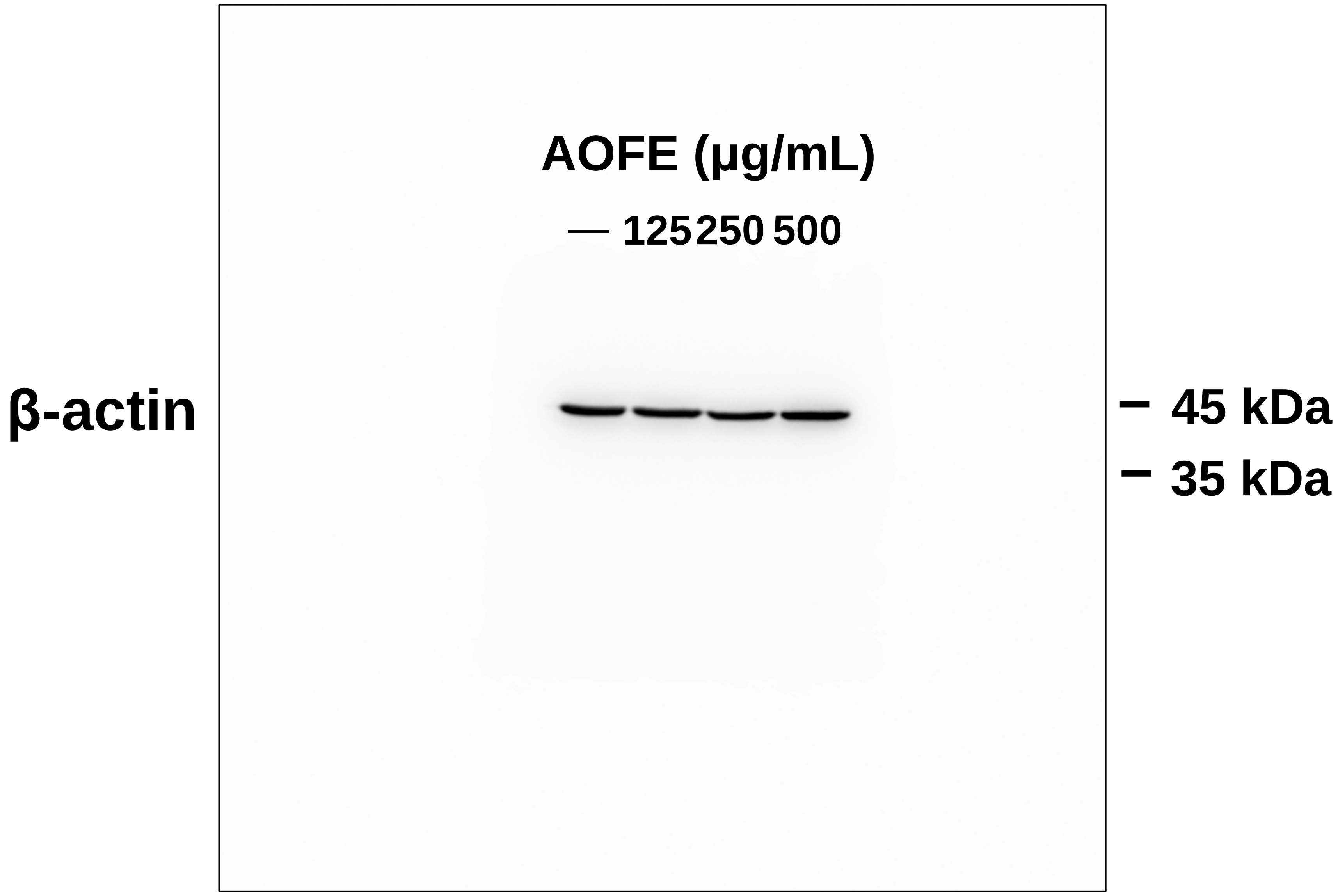

Supplementray Figure S1. Uncropped image of β-actin in figure 2A

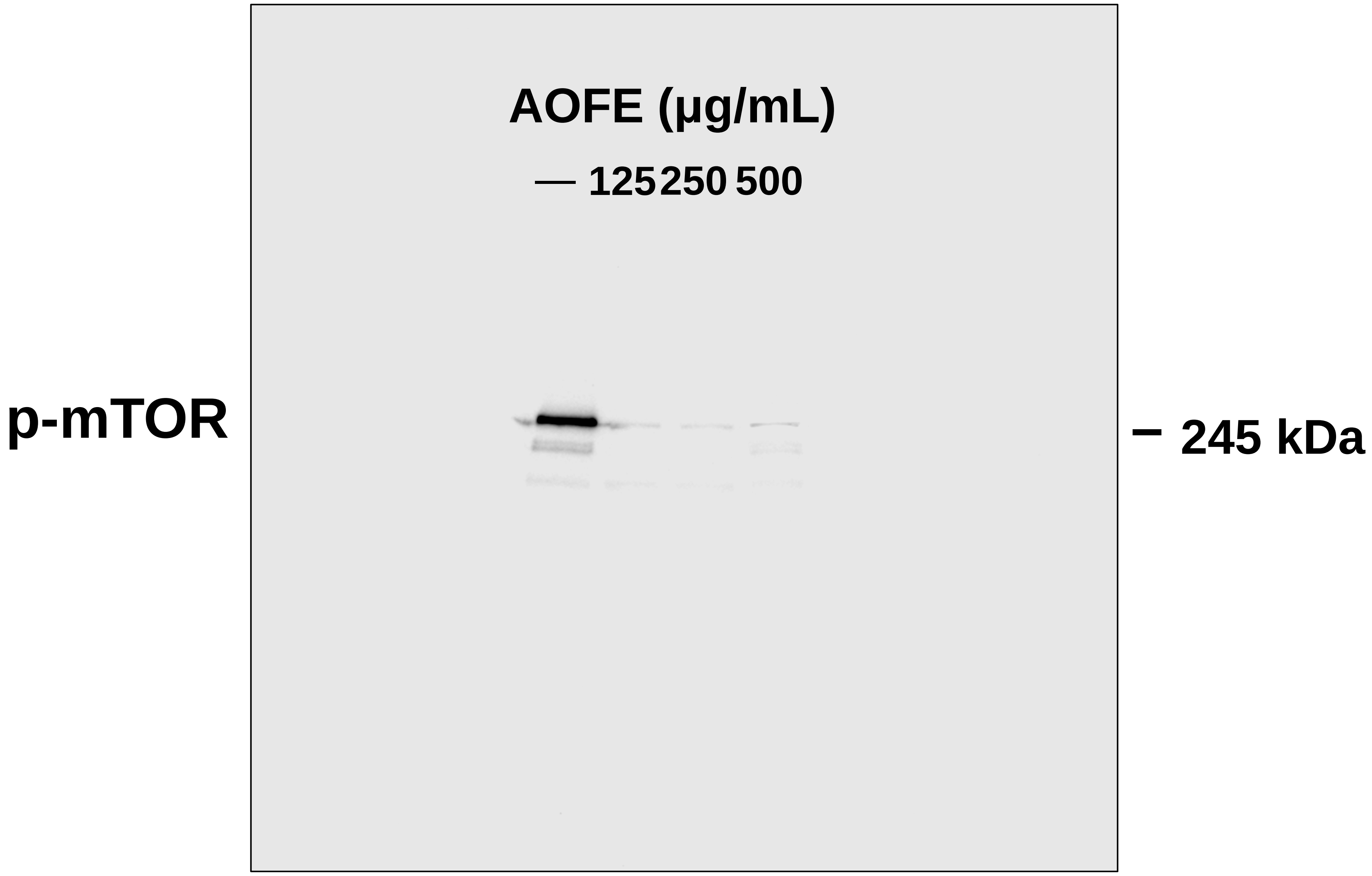

Supplementray Figure S1. Uncropped image of p-mTOR in figure 3B

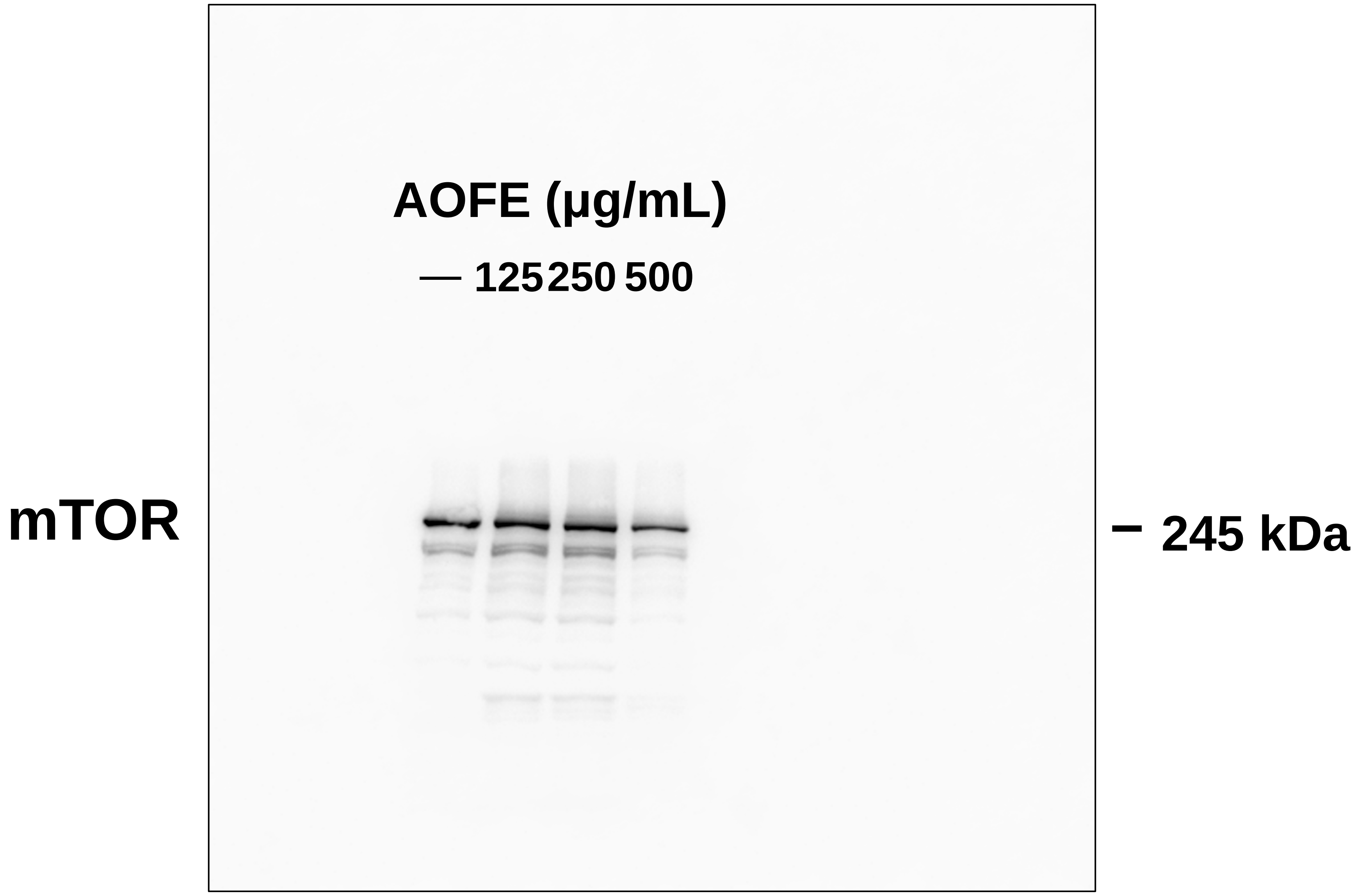

Supplementray Figure S1. Uncropped image of mTOR in figure 3B

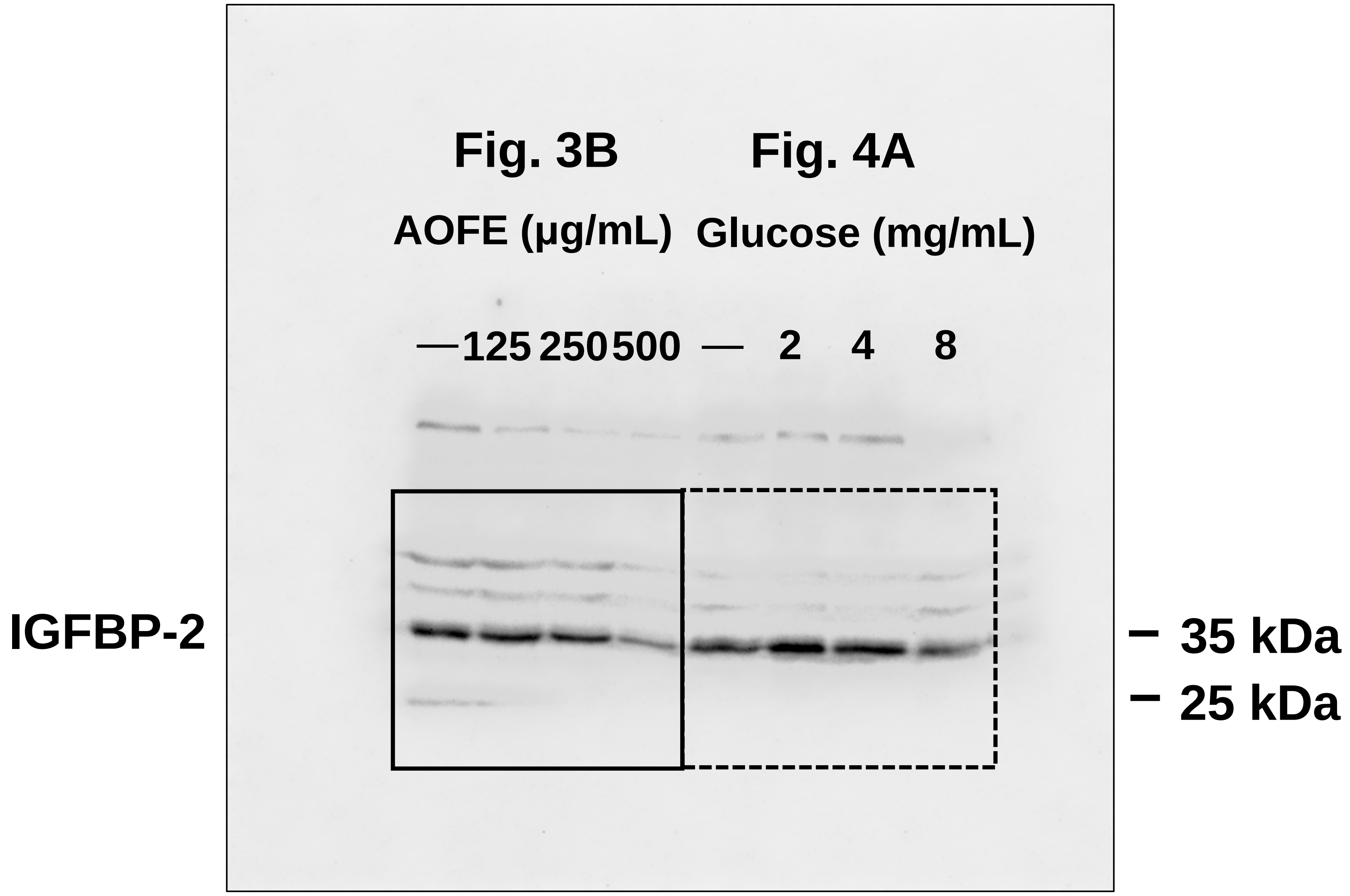

Supplementray Figure S1. Uncropped image of IGFBP-2 in figure 3B and 4A

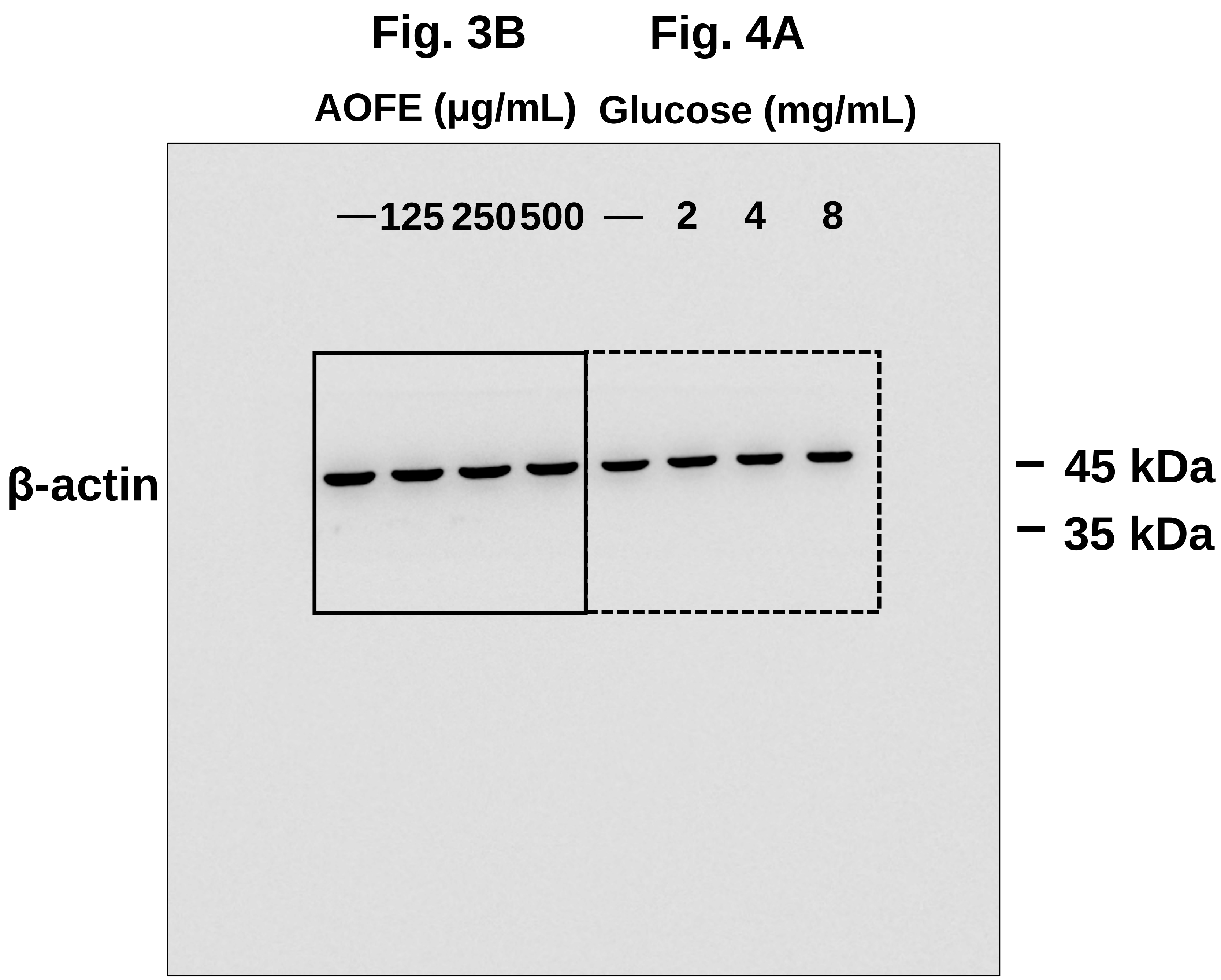

Supplementray Figure S1. Uncropped image of β-actin in figure 3B and 4A

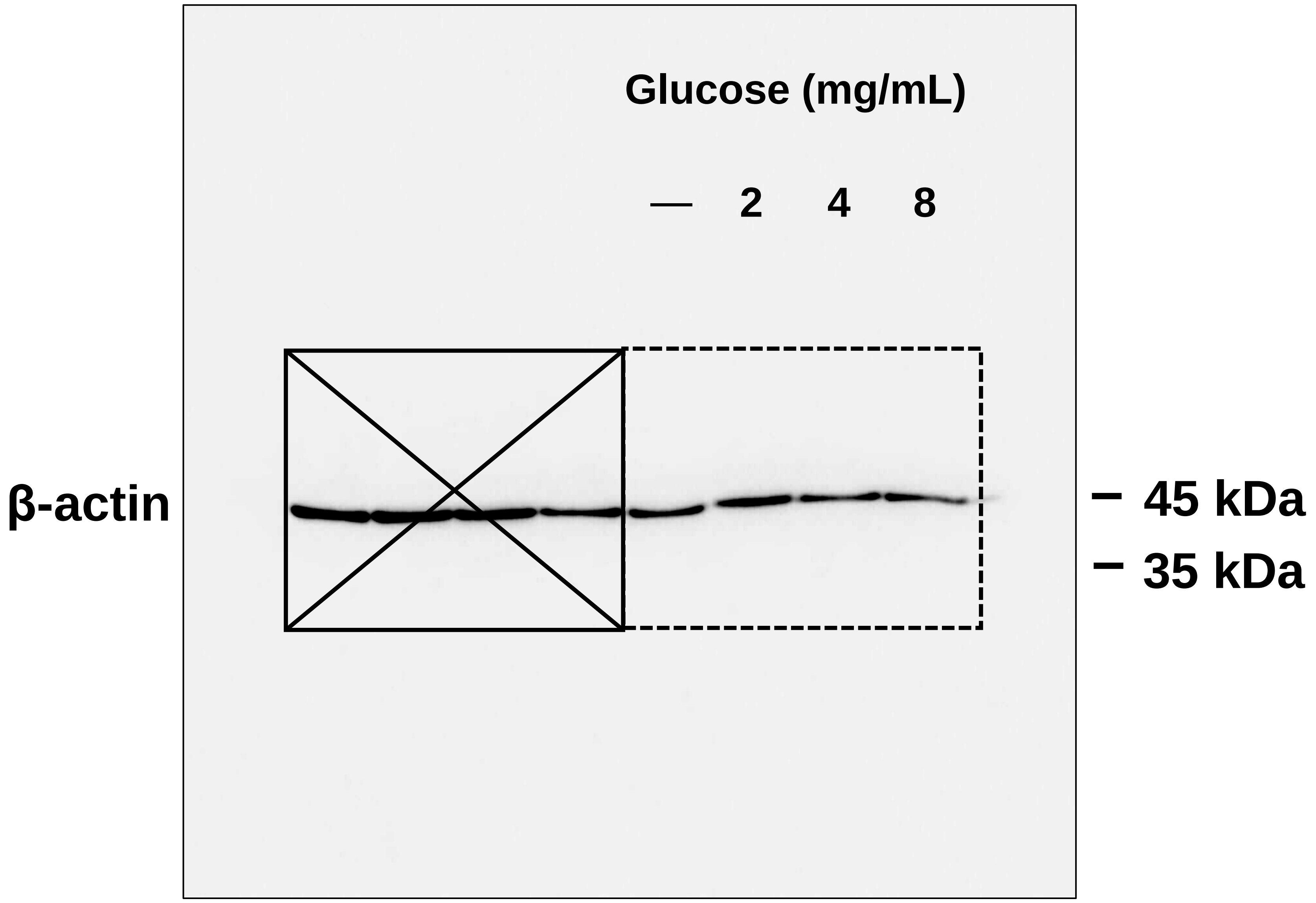

Supplementray Figure S1. Uncropped image of β-actin in figure 4A

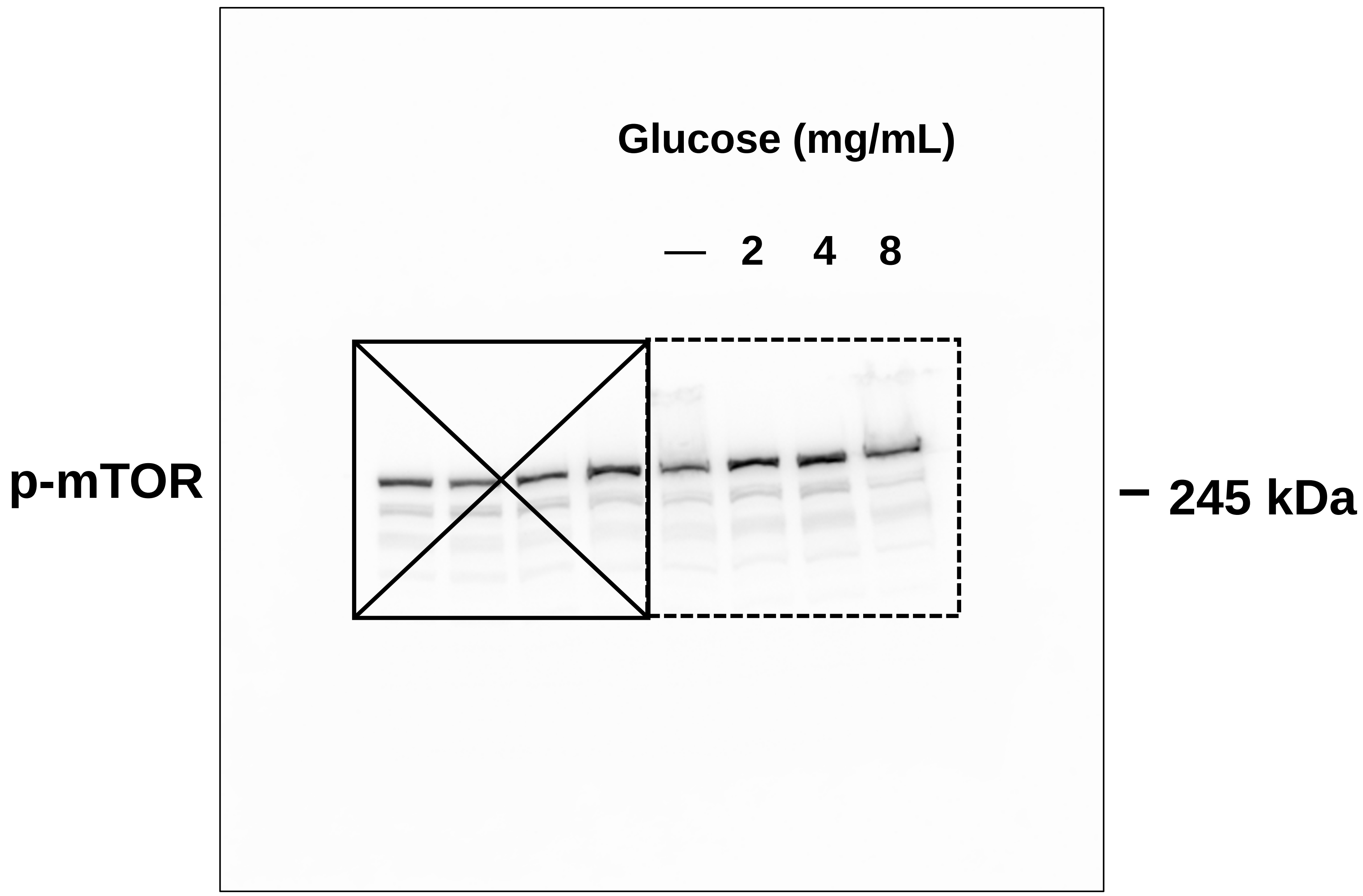

Supplementray Figure S1. Uncropped image of p-mTOR in figure 4A

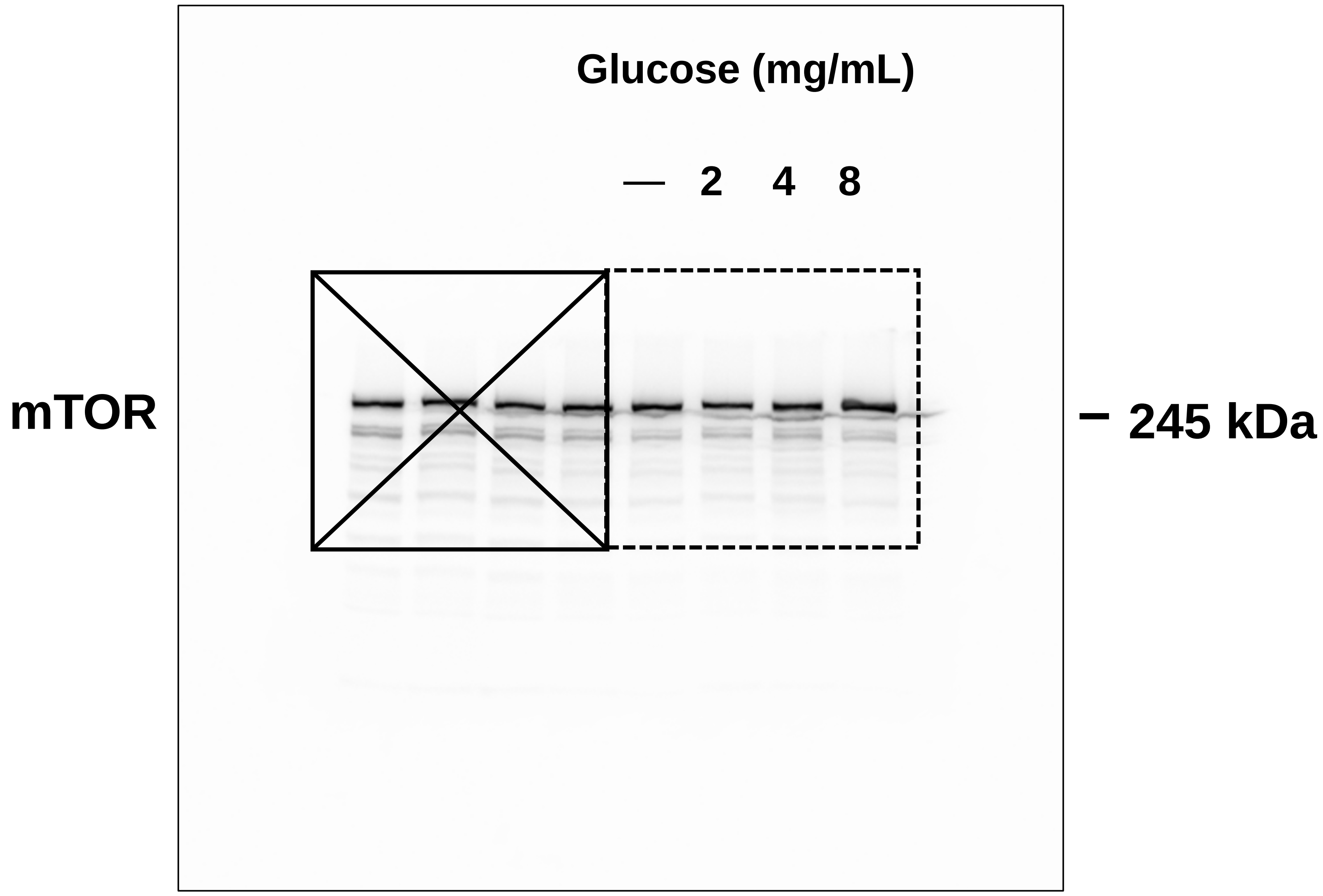

Supplementray Figure S1. Uncropped image of mTOR in figure 4A

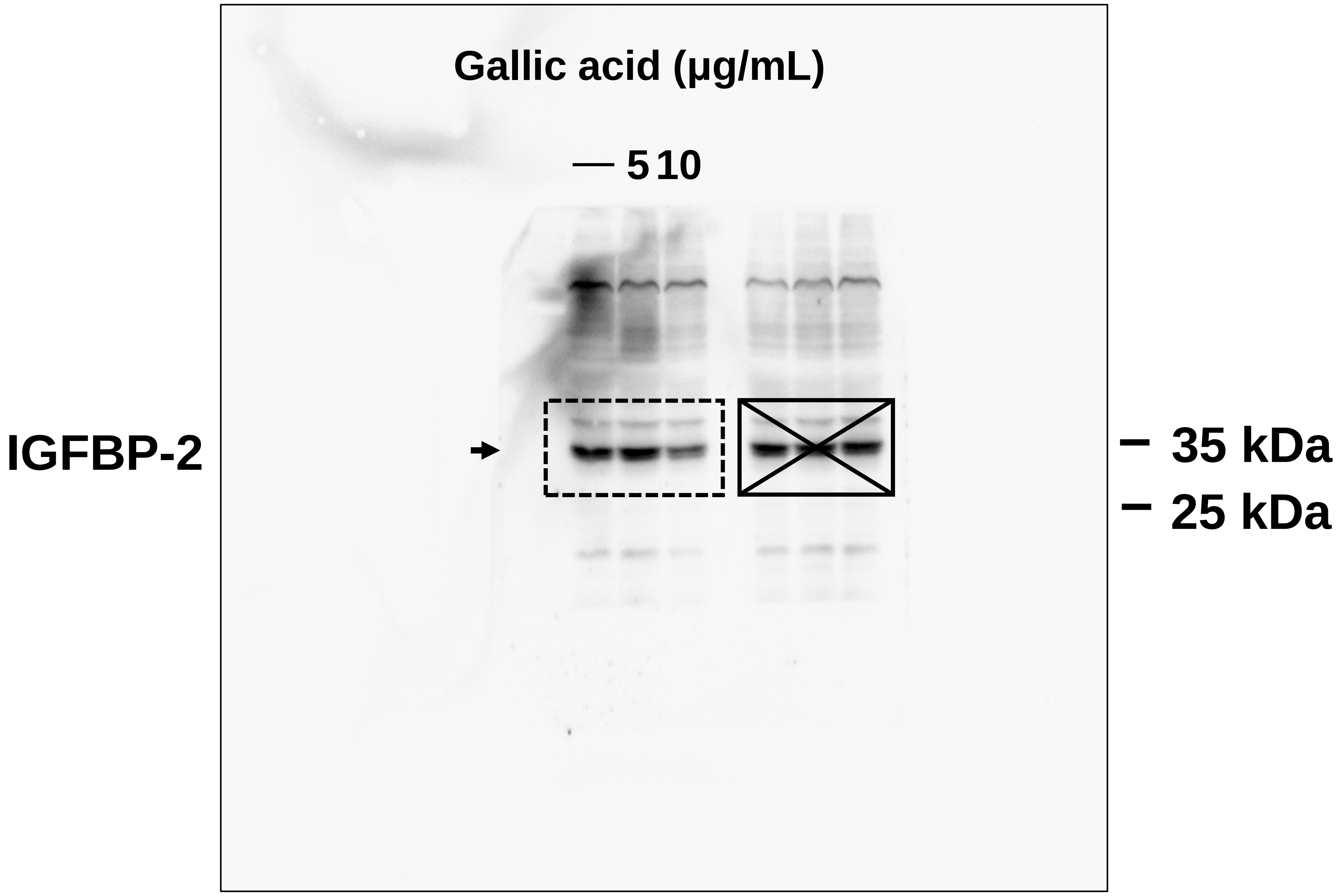

Supplementray Figure S1. Uncropped image of IGFBP-2 in figure 6C

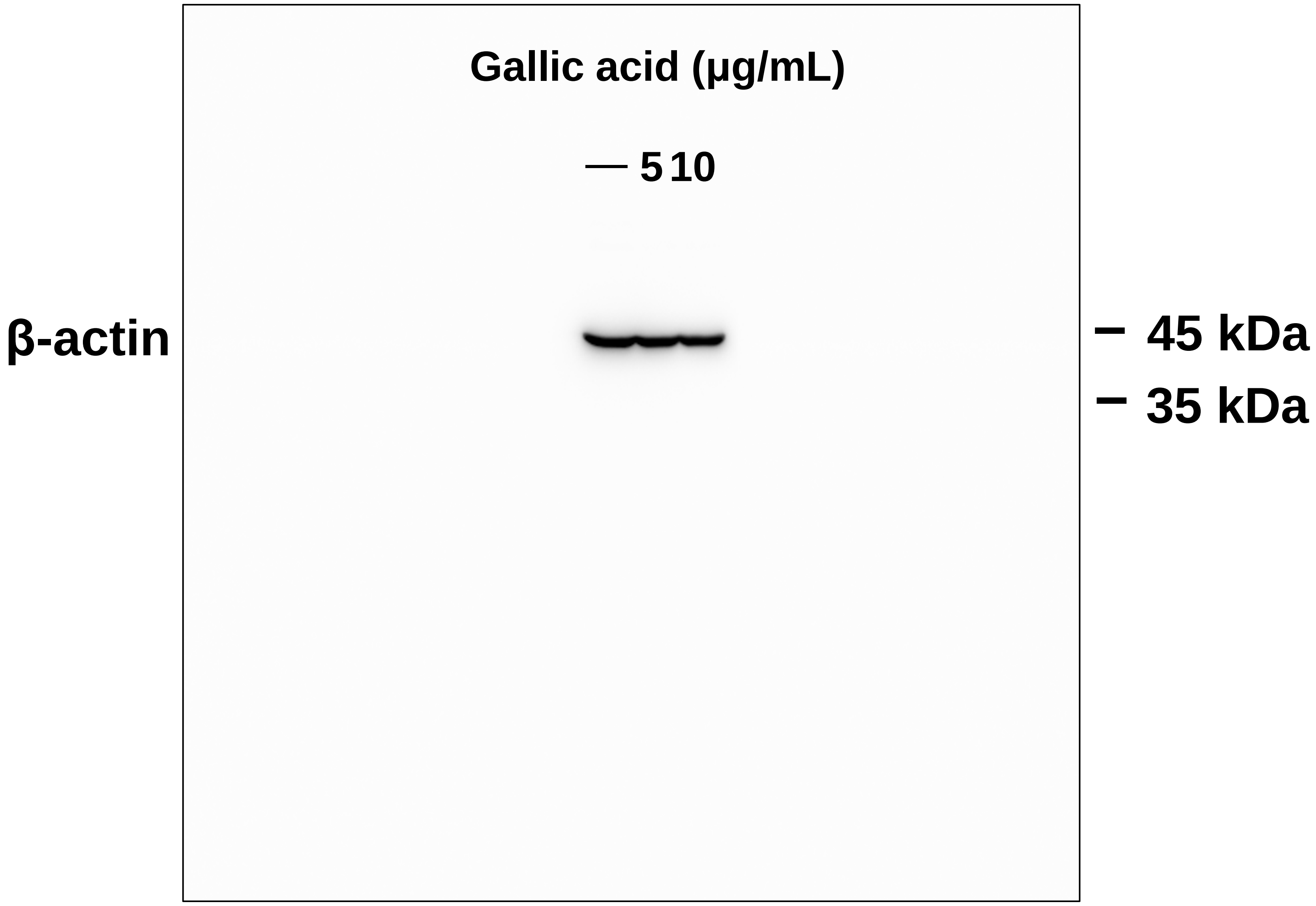

Supplementray Figure S1. Uncropped image of β-actin in figure 6C
